# Facial Paralysis Algorithm: A Tool to Infer Facial Paralysis in Awake Mice

**DOI:** 10.1523/ENEURO.0384-24.2025

**Published:** 2025-02-28

**Authors:** Elías Perrusquia Hernández, Diego Israel Villeda Arias, Claudia Daniela Montes Ángeles, Rey David Andrade González, Joel Lomelí González, Isaac Obed Pérez-Martínez

**Affiliations:** ^1^Sección de Neurobiología de las Sensaciones y Movimientos Orales, Laboratorio de Investigación Odontológica, Clínica Universitaria de Salud Integral Almaraz, Facultad de Estudios Superiores Iztacala, Universidad Nacional Autónoma de México, Cuautitlán Izcalli 54714, México; ^2^ Sección de Estudi os de Posgrado e Investigación, Escuela Superior de Medicina, Instituto Politécnico Nacional, Miguel Hidalgo, CDMX 11340, México

**Keywords:** artificial vision, facial paralysis, nerve injury

## Abstract

Facial paralysis is characterized by an injury to the facial nerve, causing the loss of the functions of the structures that it innervates, as well as changes in the motor cortex. Current models have some limitations for the study of facial paralysis, such as movement restriction, the absence of studying awake animals in behavioral contexts, and the lack of a model that fully evaluates facial movements. The development of an algorithm capable of automatically inferring facial paralysis and overcoming the existing limitations is proposed in this work. In C57/BL6J mice, we produced both irreversible and reversible facial paralysis. Video recordings were made of the faces of paralyzed mice to develop an algorithm for detecting facial paralysis applied to mice, which allows us to predict the presence of reversible and irreversible facial paralysis automatically. At the same time, the algorithm was used to track facial movement during gustatory stimulation and extracellular electrophysiological recordings in the anterolateral motor cortex (ALM). In the basal state, mice can make facial expressions, whereas the algorithm can detect this movement. Simultaneously, such movement is correlated with the activation in the ALM. In the presence of facial paralysis, the algorithm cannot detect movement. Furthermore, it predicts that the condition exists, and the neuronal activity in the cortex is affected with respect to the evolution of facial paralysis. This way, we conclude that the facial paralysis algorithm applied to mice allows for inferring the presence of experimental facial paralysis and its neuronal correlates for further studies.

## Significance Statement

Recording the faces of mice can help predict facial paralysis unbiasedly and identify the presence of a reduction in facial movement associated with injury to the facial nerve. It can also be used to study facial movements, such as facial expressions, and their neural correlates in cortical and subcortical strata. This will not only allow a deep understanding of the magnitude of the effects that facial paralysis can produce at the peripheral and central nervous system levels but also inspire further research and the search for potential treatments.

## Introduction

Facial paralysis is a condition where the facial nerve is damaged and results in the loss of function of the structures it innervates (Maspero et al., 2017; Walker et al., 2024). It has been reported that 30% of those who have facial paralysis experience long-term effects, and 5% present a high degree of sequelae after apparent recovery (Maspero et al., 2017). Damage is not limited to the muscles or other facial structures; it has also been observed in the central nervous system (CNS), in structures related to the motor and sensory response, such as the primary motor and somatosensory cortex (Klingner et al., 2011; Song et al., 2017).

Therefore, different experimental strategies have been proposed for the study of facial paralysis, mainly in rodents (rats and mice). The cardinal signs that have been used to recognize the presence of experimental facial paralysis are the movement of vibrissae, eye area, and nose orientation (Sugita et al., 1995; Severson et al., 2019; Zeng et al., 2024). However, for the application of these strategies, the use of high-cost systems is required. These proposals for analyzing experimental facial paralysis in rodents do so in animals with restricted movement, on anesthetized animals with a limited behavioral context, and with separate evaluations of the cardinal signs of facial paralysis mentioned above. Furthermore, the evaluation performed by an expert has its limitations, as it is time-consuming and is not able to detect the dynamics of facial movement (Attiah et al., 2017; Severson et al., 2019; Vajtay et al., 2019).

These methodological problems entail experimental limitations for the study of facial paralysis and its effects on the CNS, which are shown as electrophysiological changes in action potentials and dendritic retraction in the motor cortex and corticofacial structures (Urrego et al., 2011; Munera et al., 2012). Nevertheless, facial movement processing begins in premotor structures, which could be affected by facial paralysis, such as the anterolateral motor cortex (ALM), which is responsible for the planification, coordination, and execution of movements (N. Li et al., 2015), and it has also been related to the movement of facial muscles (Haiss and Schwarz, 2005).

Hence, this work attempts to use a strategy that allows the study of facial paralysis and its conditions in the CNS in awake mice and under a behavioral context. Image processing is proposed for the prediction of facial paralysis since, in recent years, it has been used to track facial movement by detecting vibrissae on the mouse face (Severson et al., 2019), to give electrophysiological tracking of orofacial movement (Syeda et al., 2024), and to detect, quantify, and classify emotional facial expressions (Dolensek et al., 2020; Le Moene and Larsson, 2023; Tanaka et al., 2023). These tools have broadly supported the study of orofacial movements and their participation at the level of the CNS. In this sense, the facial analysis proposed by Dolensek et al. (2020) can be useful for analyzing facial movement impairment in mice. Using this protocol adapted to our setup, as well as the histogram-oriented gradient (HOG) analysis from videography of facial movements, it could be possible to determine facial motor alterations after facial nerve injury.

To address this issue, we utilized two models of facial paralysis (transection or compression of the facial nerve) for the design, creation, and validation of the facial paralysis algorithm (FaPA) in mice. This rigorous process allowed us to determine the health status of the study subject, whether it was healthy, was paralyzed, or showed signs of recovery. The FaPA was specifically designed for use in awake mice with semirestricted movement, under a behavioral protocol of gustatory delivery. This allowed us to monitor facial expressions and perform extracellular electrophysiological recordings simultaneously. The FaPA's successful development and validation mark a significant step forward in the study of facial paralysis in mice.

Our results show that facial transection injury generates irreversible facial paralysis. In contrast, facial nerve compression injury generates reversible paralysis, showing disorders in whisker and facial movements in the middle and anterior parts of the mouse face. These two areas were used for the development of the FaPA, which allowed us to determine the presence of both reversible and irreversible facial paralysis. It was found to be useful for tracking facial movement during the study of facial expressions and their neural correlations in the ALM. Finally, facial paralysis causes an inability to make facial expressions and a decrease in the firing rate in ALM neurons in the irreversible model. On the other hand, in the reversible model, there is an apparent recovery of the ability to make facial expressions. Still, the neuronal activity in the ALM is not comparable with baseline values.

Therefore, the FaPA presents a practical and effective solution for studying experimental facial paralysis, with or without sensory stimuli, for orofacial movement studies, and during electrophysiological recordings at the cortex level. Its reliability and ease of use will provide researchers with a confident tool for paralysis facial studies in rodents.

## Materials and Methods

### Animals

Twelve male and female C57BL/6J mice, aged 2–6 months, were used. All procedures were carried out in accordance with the standards of the faculty ethics committee. All mice were housing at 25°C and a 12 h light/dark cycle (starting at 8:00 a.m.). Subjects were provided with standard food and water *ad libitum*. Only during conditioning and testing were mice deprived of water, allowing a maximum consumption of 4 ml per day.

### Surgery for head-fixation device implantation and craniotomy

Mice were anesthetized with isoflurane (3% for induction, 1% for maintenance). Under the surgical plane, an incision was made on the scalp, and tissues were removed to expose the skull. Using a stereotaxic system, the ALM was located 2.5 mm anterior and 1.5 mm lateral to the bregma. Two mice underwent a rectangular craniotomy with a 2 × 2 mm extension centered on the location of the ALM. The craniotomy opening was covered with dental silicone, and tissues were repositioned. All subjects had a device (made of polylactic acid) for the head-fixation system placed on the back of the skull, fixed with cyanoacrylate. Mice were given 7 d for recovery.

### Facial nerve surgery

Mice were anesthetized following the protocol, as mentioned earlier. Under the surgical plane, an incision was made posterior to the right ear of the mice. Tissues were dissected until the trunk of the facial nerve was observed. Finally, one group of mice had their nerve compressed with dissection forceps twice for 30 s with an interval of 10 s between compressions. The applied force was recorded with an Arduino-compatible compression sensor (Walfront, Model: Walfront9snmyvxw25; compression injury model). The next group underwent a complete cut of the facial nerve (transection injury model). Tissues were repositioned, and the incision was sutured. For the sham group, the facial nerve was only observed, and the incision was sutured.

### System

A semirestricted movement system was used for recording whisker movement, facial expressions, and acute neural recordings. The system consisted of a 32 cm diameter ball held by two lateral axes to an acrylic base, which allowed it only to move forward or backward. On the ball, there was a fixing bar (a bar designed and manufactured with polilactic acid and another metal) where the attachments previously implanted in the mice's heads fit.

Video recordings were obtained with a 30 Hz acquisition and 2K resolution cell phone camera (Blackview BV8800). The camera was placed perpendicular to the mouse's right face. Video recording of the whisker was made with a 120 Hz acquisition and HD resolution cell phone camera (Xiaomi Note 8). The camera was placed on the superior part of the mouse's face and allowed the recording of the right and left whiskers.

### Facial paralysis model evaluation

Nine mice were used: three with facial nerve transection, three with compression, and three sham mice. Twenty-three measurements were made: one on Day −1 (baseline); three more at 0.5, 6, and 24 h after the injury, respectively; and finally, one per day from Days 2 to 20 after the injury. Whisker movement was measured for 2 min (considered a session), without stimulation, through the angulations formed by the vibrissae with its insertion in the whiskerpad. The initial position of the whisker was considered as 0°; from the initial position, subsequent angulations were obtained, forming a continuous signal. The minimum angulations were located throughout the signal. From each minimum to the minimum value, a whisker movement cycle was considered. This consisted of protraction movement, moving from the most posterior position of the whisker (minimum angle) to the most anterior (maximum angle), and retraction, moving in the opposite direction to the previous movement. Each movement cycle respected a certain amplitude, which was obtained by subtracting the minimum angle from the maximum. The average amplitude of all the cycles in 2 s was taken as the threshold. Above this threshold, it was considered as a long amplitude, and below this threshold, it was considered as a short amplitude. Each movement cycle was considered 1 Hz, and all the movement cycles throughout the 2 min of the video recording were used. Time window was chosen as it was sufficient to be able to discriminate between basal and moving states in the recorded whiskers, as described below and in the Results section.

### Whisker movement tracking

For the study of whisker movement, acrylic paint was used to mark a whisker from both the ipsi- and contralateral sides of the lesion. Video recording was transformed into frames and processed with MATLAB. The initial step of processing was to identify the color of the whisker painted in each frame. Then, the identity of the pixels with this color was obtained. The image was binarized, where the pixels belonging to the whisker were replaced by white, whereas the rest were black. MATLAB Steel function was used to clean the detected area, which allowed us to eliminate pixels outside the required area (within a range of 5 pixels around). Finally, with the angle function, the angulation of the whisker was obtained, considering the reference point indicated and previously described. The smallest angle detected during the video was always considered 0°.

### Facial motion analysis

To study facial expressions and movement, the HOG image descriptor was used in each frame using the following parameters: oriented histogram content, 8; cell box length size, 32 pixels, and cell per block, 1, along with a previous normalization processing using the power compression law. For the study of facial paralysis, mice face image was cut into three zones: anterior, middle, and posterior zones. This was done over 2 min of evaluation; this is because facial paralysis can be observed (through whisker movement) in this time window (as described in the Results section). Three thousand six hundred frames were obtained per zone for the study of facial expressions. An intrinsic state (pleasure and disgust) and a neutral state (water) were studied 5 s before (control) and after oral stimulation were analyzed, which corresponded to a total of 300 frames. Each frame was transformed from RGB to grayscale, and the mathematical vectors (HOGs) were subsequently obtained. All videos were processed with MATLAB. We used the MATLAB *cpselect* and *imwarp* functions to align the frames extracted from each video to the video used for the creation of the pleasant emotional prototype (described later), with the aim of avoiding existing variations between videos. Each video was aligned with a baseline video. Four anatomical points, two in the eye and two in the nose, were considered as references for the alignment. Using the *imagetform* function and considering the anatomical points, the video was rotated to the position of the baseline video until it was aligned. In this way, the variation between each video recording was reduced, and comparison between them was made possible.

### FaPA design

The computational algorithm (CA) for inferring facial paralysis is a bimodal decision system that indicates whether the condition exists or not. To do this, the movement threshold of different sections of the face (anterior, middle, and posterior areas) is considered. The threshold is obtained from mice with facial paralysis by transection and is determined by the sum of the average of the difference between HOGs and their standard deviation:

Diff(x)=Frame(1)−Frame(x),

where Diff is the difference between frames and Frame is the frame converted into HOG mathematical vector:

$$\mathrm{mean}=\frac{\sum_{i=1}^{\mathrm{length}(\mathrm{Diff})}\;\mathrm{Diff}(i)}{\mathrm{length}(\mathrm{Diff})},$$

where mean is the average of differences between frames and *i* is the evaluations (23, including the baseline):

$$\mathrm{std}=\sqrt{\frac{\sum_{i=1}^{\mathrm{length}(\mathrm{Diff})}\;\mathrm{Diff}(i)}{\mathrm{length}(\mathrm{Diff})-1}},$$

where std is the standard deviation of the difference between frames:

Threshold=mean+std.

To determine the presence of facial paralysis, different variables were considered: areas of the face, average threshold per group or individual, and the normalization of the data. In this way, 10 facial paralysis decision models were obtained. To decide if there is facial paralysis, values must be lower than the threshold of the different areas. If this occurs, the value is determined with a binary value of 1. Otherwise, the value is 0:

Model1=LowI(A)vLowI(M),

Model2=LowGN(A)+LowGN(M),

Model3=LowI(A)+LowI(M),

Model4=LowG(A)vLowG(M),

Model5=LowG(A)+LowG(M),

Model6=LowI(A)vLowI(M)vLowI(P),

Model7=LowI(A)+LowI(M)+LowI(P),

Model8=LowG(A)vLowG(M)vLowG(P),

Model9=LowG(A)+LowG(M)+LowG(P),

Model10=TotalLowdiff,

where LowG is the binary values 1 (below threshold) and 0 (above threshold), where the threshold is averaged per group; LowGN is the binary values 1 (below threshold) and 0 (above threshold), where the threshold is averaged per group and data to obtain threshold and binary values are normalized; LowI is the binary values 1 (below threshold) and 0 (above threshold), where threshold is individual per mouse; *A* is the anterior zone; *M* is the middle zone; *P* is the posterior zone; and *N* is the mice per group (*n* = 3).

Subsequently, an attempt was made to extract the threshold individually for mice injured by transection and compression. To do this, mice injured by transection were used, where the threshold for each mouse was obtained, considering the average difference between frames:

Diff(x)=Frame(1)−Frame(x),

where Diff is the difference between frames and Frame is the frame converted into HOG mathematical vector:

$$\mathrm{Threshold}=\frac{\sum_{i=1}^{\mathrm{length}(\mathrm{Diff})}\;\mathrm{Diff}(i)}{\mathrm{length}(\mathrm{Diff})},$$

where Threshold is the average of the differences between frames and *i* is the evaluations (23, including the baseline).

Once the threshold was created, the number of evaluations for the optimal functioning of the algorithm was sought:

$$\mathrm{Threshold}(x)=\frac{\sum_{i=2}^{\mathrm{length}(\mathrm{Diff}-x)}\;\mathrm{Diff}(i)}{\mathrm{length}(\mathrm{Diff})},$$

where *x* is the number of tests (up to 22).

It was determined that two assessments, one at baseline and one at a standstill, were sufficient to obtain an appropriate threshold. Finally, the assessment that would be ideal for the algorithm's functioning was sought:

$$\mathrm{Threshold}(x+1)=\frac{\mathrm{Diff}(1)+\mathrm{Diff}(x)}{2}.$$

This technique is used for mice individually for the transection and compression injury groups with the previously used model:

Model2=LowGN(A)+LowGN(M).

As a last resort, the algorithm was used to track movement in real time:

Movement(Frame)=LowGN(A(Frame))+LowGN(M(Frame)),

where Movement is the movement detected by the algorithm. If the value is <2, there is movement; otherwise, there is no movement.

If the percentage of moments without movement is >90%, the system concludes that the mouse is paralyzed.

To conclude that the system is correctly predicting facial paralysis, the decision-making percentage was calculated, which consists of the percentage of days that the system detects facial paralysis with respect to the days in which facial paralysis exists. For this, the whisker movement analysis is considered; the days in which there is facial paralysis are those in which the whisker movement has statistically significant differences compared with the baseline evaluation.

### Gustatory stimulation test

A behavioral task adapted from Dolensek et al. (2020) was used to analyze the facial movement. The behavior assay consisted of the release of sucrose (20%), quinine (0.3 mM), and regular water directly into the mouth of the mice through an intraoral cannula. Mice were placed in the semirestricted head system, recording the walk (speed at which the mouse moved on the system) and facial expressions. A 5 min baseline recording was made, in which no stimulus was presented. Further, the video recording was made during oral stimulation. For the release of a solution, mice must avoid walking for 10 s. When this condition was met, a semirandomly chosen solution was delivered, eight drops of 4 μl each, and a facial video recording was made for 1 min. This was considered a trial. For a new trial to start, the condition of not walking for 10 s had to be met, and a session consisted of five trials per solution. Finally, a 5 min video recording was made with the solution infusion pump activated without releasing any solution. For the electrophysiological recordings, the previous protocol was repeated. However, stimulation was performed only with sucrose.

### Electrophysiology

**Figure 1.**
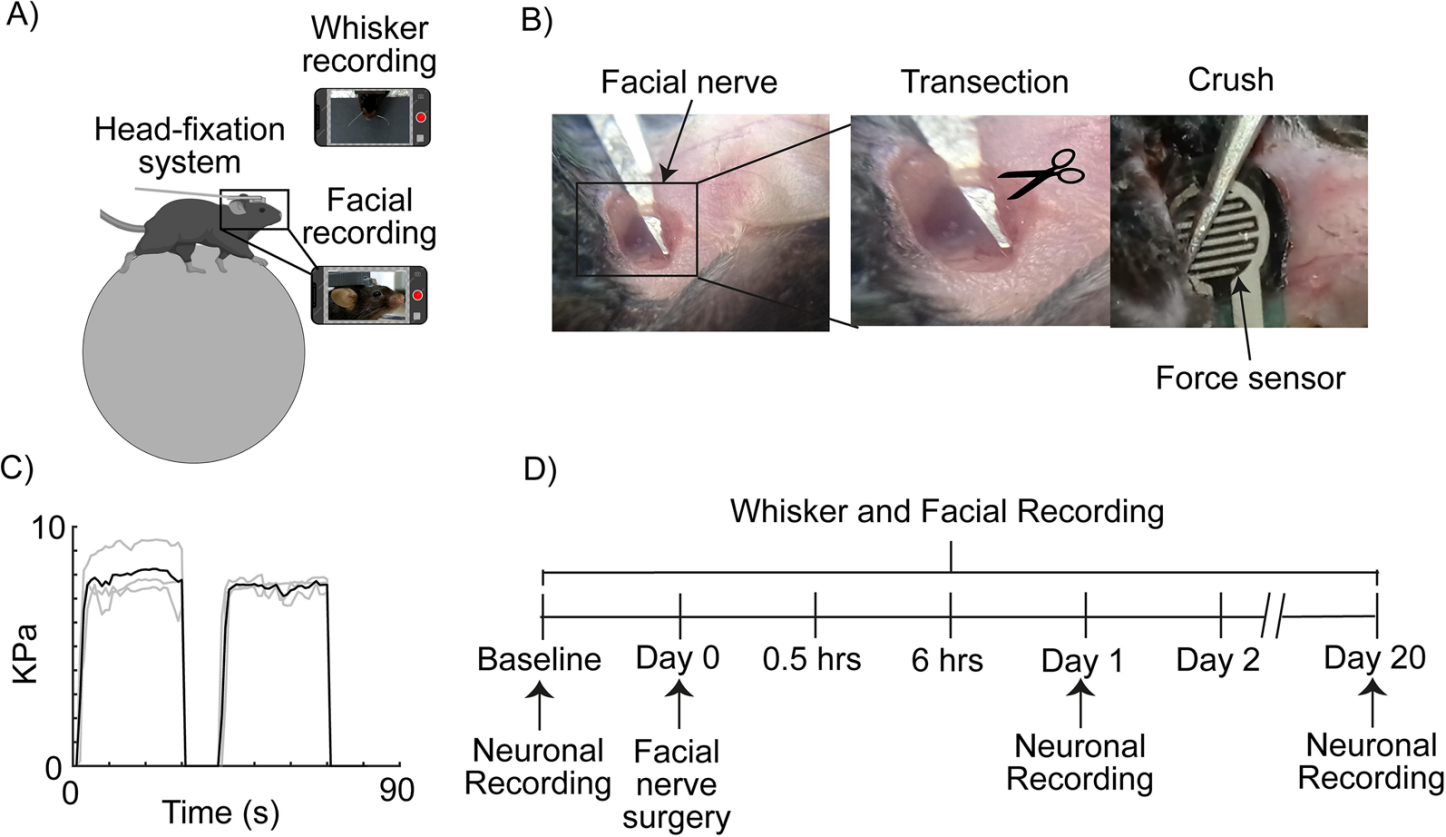
Experimental design. ***A***, Schematic of the semirestricted movement system and simultaneous video recording of whiskers and the face of the mouse. ***B***, Surgical process of facial paralysis models of nerve injury by transection and compression. ***C***, Monitoring of the force applied to the facial nerve when it is compressed; the gray lines represent each mouse (*n* = 3) and the black line average (*p* ≤ 0.05 one-way ANOVA test). ***D***, Experimental timeline. Detailed statistics in Extended Data Table 1-1.

A 16-channel microelectrode array (Tucker-Davis Technologies, OMN1030-16) was acutely implanted in CMAL (Fig. 1). Microelectrode voltage signals were acquired, digitalized, and filtered with an Open Ephys multichannel acquisition processor. Only individual neurons with action potentials with signal-to-noise ratios greater than 3:1 were selected for analysis and sorted using the Plexon Offline Spike-Sorter system.

10.1523/ENEURO.0384-24.2025.t1-1 Table 1-1 **Statistical details in the forces applied in the facial nerve.** Difference between the forces applied in facial nerve in the crush group (mouse 1 vs. mouse 2 vs mouse 3; Figure 1C). Significance level p<=0.05. Download Table 1-1, RTF file.

### Creation of the facial prototype

Based on the analysis proposed by Dolensek et al. (2020), a basal or resting state facial prototype was determined, which was created using frames of a mouse during the period prior to oral stimulation (250 frames), transformed into HOGs, and averaged. This basal prototype in HOGs was compared with the entire stimulation set with sucrose, quinine, and water, calculating Pearson’s correlation coefficient. From each solution, the 10 frames with the greatest difference with the basal facial prototype (those with the lowest correlation coefficient) were taken. The average of these frames resulted in a single HOG considered as the emotional prototype (pleasant for sucrose, disgust for quinine, and neutral for water).

### Statistical analysis

The comparison between the values obtained by the force sensor when compressing the facial nerve was made with a one-way ANOVA analysis, comparing the applied force between the three subjects in the group. During the tracking of the whisker movement, *ischange* function was used to detect the moment with the greatest change in a signal, which includes the following values:

C(A1)+C(A2)+t<C(A).

where *A* is the data vector (whisker movement signal), *A*1 is the first segment of the vector, *A*2 is the second segment of the vector, *C* is the cost function, and *t* is the threshold created by the MATLAB threshold function

The threshold function creates an object of discrete state threshold transitions and is specified by the mean transition levels. The cost function tells us how close a segment is to this segment.

After that, the amplitudes before and after the change point were considered and were analyzed with Student’s *t* test analysis. The area under the curve of the whisker movement cycle of the 23 evaluations performed (baseline, 30 min, 6 h, and from Days 1 to 20 after the facial injury) was obtained and analyzed with one-way ANOVA and Tukey’s post hoc test. To study the proportion of long and short whisker movement amplitudes, a chi-square goodness-of-fit analysis was used. Whisker movement frequency values were organized and graphed using the MATLAB spectrogram function.

During the tracking of mouse facial movement in the three facial areas, a one-way ANOVA analysis with Tukey’s post hoc test was used. Pearson’s correlation analysis was applied between the whisker movement amplitudes and the difference between the HOG of the mice's faces. For the analysis of facial expression, the frames before and after the release of the different solutions were used; using the MATLAB cluster-gram function, an automatic hierarchical tree clustering was performed. A t-distribution stochastic neighbor analysis was performed for the clustering of frames associated with a particular solution. Finally, Pearson’s correlation analysis was performed between the facial prototype (previously described) and the frames before and during oral stimulation.

To obtain the accuracy of the system, the value *d*′ was used, which considers the following:

$$d^{\prime }=z(\mathrm{FA})-z(\mathrm{AC}),$$

where AC is the hit (the algorithm infers facial paralysis after transection facial injury), FA is the false alarm (there is no facial paralysis, and the algorithm does infer it), and *z* is the typical *z* value.

For the electrophysiological analysis, a Wilcoxon rank sum analysis was performed for the population neuronal activity with data before and after oral stimulation with sucrose, activation of the solution infusion pump, and at the time of starting to walk. The neuronal activity consists of the average of the *z* value (normalized in a range of −2 to 2) obtained from the peristimulus time histogram (PSTH), which shows us the amount of activation of the neurons recorded in an interval of 100 ms. The *z* value was obtained with the following formula:

$$\mathrm{zscore}=\frac{\left(\mathrm{PSTH}-m\right)}{\mathrm{std}},$$

where PSTH is the neuronal activity, *m* is the mean obtained from the neuronal activity prior to stimulation (10 s), and std is the standard deviation obtained from the neuronal activity prior to stimulation (10 s).

Finally, a cross-variance analysis was used for the relationship between neuronal activity, facial expression, and facial movement. The cross-variance values between neuronal activity with facial expression and neuronal activity with facial movement were analyzed with Pearson’s correlation. For more details on the *p* values of each statistical test, see attached tables.

### Code accessibility

The code/software described in the article is open source and is freely available on GitHub (https://github.com/FaPA305/FaPA-Facial-Paralysis-Algorithm-a-tool-applied-in-mice). The code is also available in Extended Data 1. FaPA can be used in MATLAB software (version 2023b with image processing toolbox). The specification of a computer is anyone that can run MATLAB software.

10.1523/ENEURO.0384-24.2025.d1 Extended Data 1 Extended data includes a copy of the GitHub repository. It contains a copy of code that charges videos (baseline and transection), processes videos, extracts HOG vectors from videos, calculates a threshold to detect facial paralysis, and applies the FaPA algorithm to both videos. Download Extended Data 1, PDF file.

## Results

### Reversible and irreversible facial paralysis model in mice

To determine the presence of facial paralysis in the study subjects, a semirestricted movement system and bilateral video recording of their whiskers were used (Fig. 1*A*). Two models of facial paralysis were used: transection and compression. In addition to these, a sham group was added as a control group (Fig. 1*B*). For the transection injury to be uniformly performed in all subjects, a complete cut of the nervous tissue was made, and the loss of continuity of the facial nerve was confirmed. For the compression injury, a force sensor was used (see Materials and Methods), which measured the applied pressure on the nervous tissue (Fig. 1*B*). This way, the measurements obtained in each subject did not present statistically significant differences (Fig. 1*C*), which indicated that the injuries were comparable with each other. All experiments were performed according to the timeline described in Figure 1*D*.

To study the effects of facial paralysis, a video recording of whisker movement was used. A whisker was tracked both ipsi- and contralateral to the injury. Its position was determined for each frame of the video. The position was given by the angle formed between the whisker and its insertion point in the whiskerpad. The accumulation of the whisker positions throughout the video recording represented a signal. Using a mathematical model (MATLAB function: *ischange*), which allowed us to determine abrupt changes in a signal, we searched for the frame where the whisker had the greatest changes in its angulations, which we called the change point (Extended Data Fig. 2-1*A,B*). The minimum and maximum peaks of the signal were detected 2 s before and after the change point (Extended Data Fig. 2-1*B*); this was used to determine the amplitude of the whisker movement, which consisted of the subtraction between the angle of the most anterior position (minimum peak) and the posterior position (maximum peak). With the above, a difference can be observed between the amplitudes of the movement before and after the point of change, prior to the facial injury for the transection, compression, and sham groups (Extended Data Fig. 2-1*C*). This showed us the change between the resting state of the whisker and the moment in which movement began in all groups. In this way, only the moments in which there was whisker movement were analyzed.

**Figure 2.**
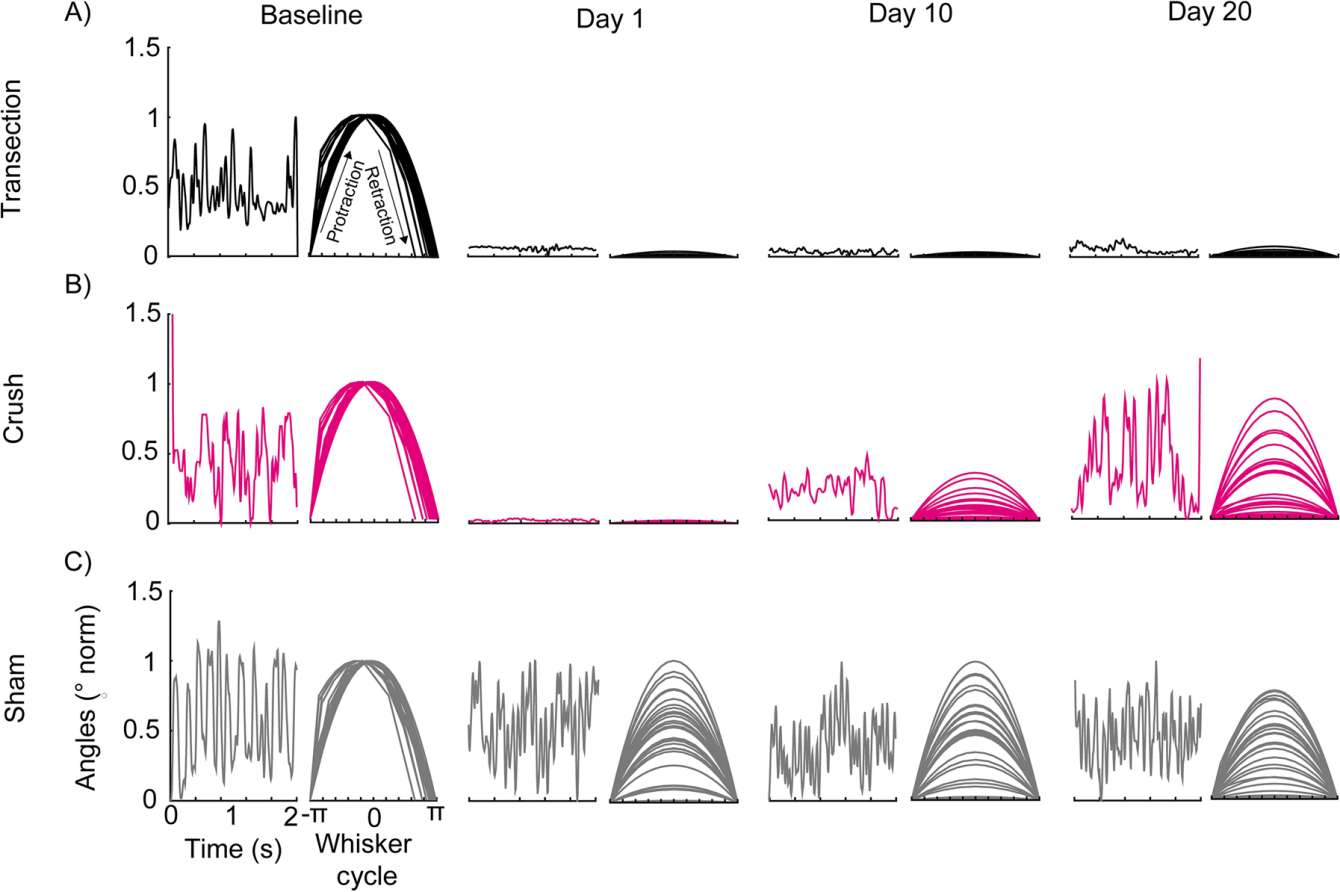
Effect of facial paralysis on whisker movement cycle. Whisker movement monitoring of a single mouse per group (***A***) transection, (***B***) crush, and (***C***) sham, prior to surgery and on Days 1, 10, and 20 postoperatively. The graphs on the left side of each column represent the dynamics of whisker movement over 2 s of evaluation, and on the right, their movement cycle is shown. Complementary information is shown in Extended Data Figures 2-1–2-2 and Table 2-1.

**Figure 3.**
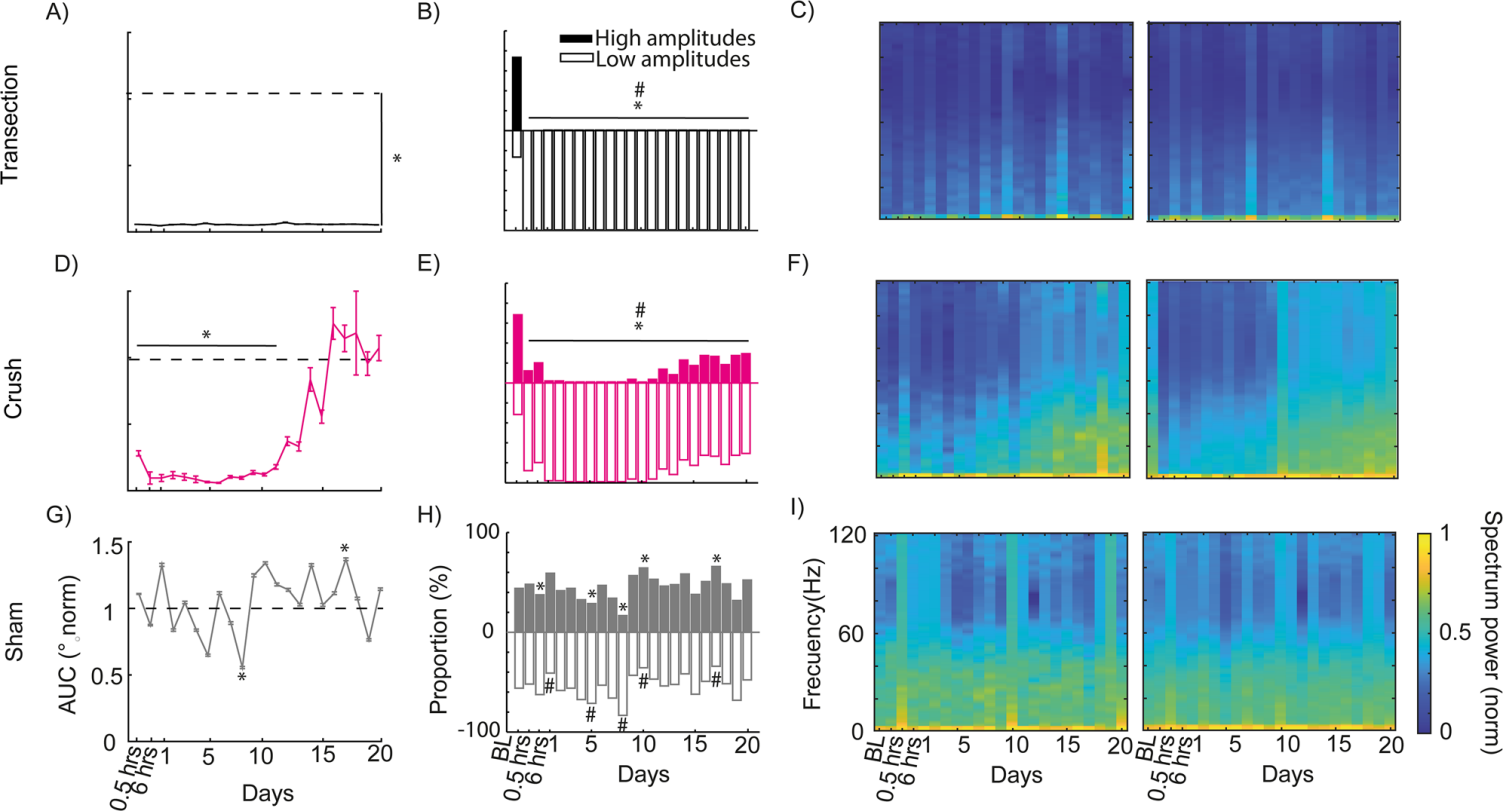
Physiological changes in whisker movement after facial paralysis. ***A***, Area under the curve of the whisker movement cycle assessed from half an hour to Day 20 after surgery (baseline vs lesion *p  ≤  0.05 one-way ANOVA test, Tukey’s test *p* ≤ 0.05, *n* = 3). ***B***, Proportion of high and low amplitudes obtained from the whisker movement cycle (baseline vs lesion in high amplitudes *p ≤ 0.05 and baseline vs lesion in low amplitudes #*p* ≤ 0.05 chi-square test). ***C***, Spectrogram of whisker movement frequency over 2 s; the left graph represents one mouse and the right the average of the transection injury group. This description is repeated in ***D*** (baseline vs lesion *p ≤ 0.05 one-way ANOVA test, Tukey’s test *p* ≤ 0.05), ***E*** (baseline vs lesion in high amplitudes **p ≤ 0.05 and baseline vs lesion in low amplitudes ##*p* ≤ 0. 05 chi-square test), and ***F*** for the compression injury group (*n* = 3) and ***G*** (baseline vs surgery *p ≤ 0.05 one-way ANOVA test, Tukey’s test *p* ≤ 0.05), ***H*** (baseline vs lesion in high amplitudes *p ≤ 0.05 and baseline vs lesion in low amplitudes #*p* ≤ 0.05 chi-square test), and ***I*** for the sham group (*n* = 3). The dotted lines in ***A***, ***D***, and ***G*** represent the baseline. Detailed statistics in Extended Data Tables 3-1–3-4.

Whisker movement cycle, given by the protraction movement (travel from the most posterior position to the most anterior position of the mouse's face) and retraction (travel from the most anterior position to the most posterior position of the mouse's face), was used to study the degree of mobility that whiskers had. The area under the curve of this cycle was used to determine the movement width, both before and after nerve injury (Fig. 2). During baseline evaluation, the three groups (transection, compression, and sham) behaved in a similar way, where the whiskers respected retraction and protraction movement (Fig. 2*A-C*). Following nerve injury by transection, the whisker movement cycle was altered, showing a decrease in the amplitude of movement from half an hour to Day 20 after the injury (Figs. 2*A*, 3*A*). The compression injury showed similar effects to those of transection during the first 9 d. Subsequently, an increase in the amplitudes of the whisker movement cycle was observed until reaching baseline values from Days 15 to 20 after the injury (Figs. 2*B*, 3*D*). The sham group maintained the amplitudes of its movement cycles throughout the evaluations, showing a decrease in their amplitudes on Days 8 and 17 after surgery (Figs. 2*C*, 3*G*). The opposite occurred on the contralateral side of the injury: in the transection and compression groups, the whisker movement cycle or its amplitudes were not lost (Extended Data Fig. 2-2). This showed that the transection injury generates irreversible facial paralysis since the whisker mobility was never recovered. Compression injury is reversible since an apparent recovery of function is observed from Day 15, and this is directly due to the damage to the facial nerve and not to tissue manipulation.

10.1523/ENEURO.0384-24.2025.f2-2 Figure 2-2 **The cycle of whisker movement contralateral to facial injury.** Whisker movement was monitored in one mouse per group, (A) transection and (B) crush, prior to surgery and on days 1, 10, and 20 postoperatively. The graphs on the left of each column represent the tracking of whisker movement dynamics over 2 seconds of assessment, and the movement cycle is shown on the right. Download Figure 2-2, TIF file.

10.1523/ENEURO.0384-24.2025.f2-1 Figure 2-1 **Detection of the onset moment of whisker movement.** A) Tracking of a whisker movement over 4 seconds. The green line shows the moments of greatest change in the signal. B) Detection of the high points of the signal (peaks) marked by the blue circles; the green line represents the moments of abrupt changes along the signal, and the black point shows the point with the greatest change (change point). C) Amplitudes formed in the signal before and after the change point for the transection group, in the upper panel (t-test * p<=0.05); compression, in the middle panel (t-test * p<=0.05) and sham, in the lower panel (t-test * p<=0.05). Detailed statistics in Extended Data Table 2-1. Download Figure 2-1, TIF file.

10.1523/ENEURO.0384-24.2025.t2-1 Table 2-1 **Statistical details in whisker movement.** Difference between the amplitudes before and after the change point in transection, crush, and sham groups (Figure 2-1C). Significance level p<=0.05. Download Table 2-1, RTF file.

### Physiological differences in whisker movement between transection and compression facial paralysis models

The first physiological aspect to be studied was the movement amplitude, which showed us the range of movement that the whisker is capable of. In transection facial injury, there was no wide movement of the whisker, and this was observed throughout the 20 d of assessment (Fig. 3*A*). However, whisker movement did not have a single amplitude; therefore, all amplitudes were averaged over 2 s of evaluation. Any amplitude above the average was considered a long amplitude and below a short amplitude. Prior to the injury, long and short amplitudes are shown, which changed their distribution after the injury, where short amplitudes predominated (Fig. 3*B*). Finally, the frequency of movement was obtained, where the prevalence of low frequencies (0–5 Hz) after the facial injury was observed (Fig. 3*C*). This showed that irreversible facial paralysis not only causes a loss in the dynamics and range of whisker movement but also affects the frequency at which it is performed.

10.1523/ENEURO.0384-24.2025.t3-1 Table 3-1 **Statistical details in whisker movement with facial paralysis.** Difference in area under the curve between baseline vs. days post facial paralysis in transection, crush, and sham groups (Figure 3A, Figure 3D and Figure 3G). Significance level p<=0.05. Download Table 3-1, RTF file.

10.1523/ENEURO.0384-24.2025.t3-2 Table 3-2 **Statistical details in the proportion of high and low amplitudes in transection group.** Difference between the baseline day vs. days post facial paralysis (Figure 3B). Significance level p<=0.05. Download Table 3-2, RTF file.

10.1523/ENEURO.0384-24.2025.t3-3 Table 3-3 **Statistical details in the proportion of high and low amplitudes in the crush group.** Difference between the baseline day vs. days post facial paralysis (Figure 3E). Significance level p<=0.05. Download Table 3-3, RTF file.

10.1523/ENEURO.0384-24.2025.t3-4 Table 3-4 **Statistical details in the proportion of high and low amplitudes in the sham group.** Difference between the baseline day vs. days post facial paralysis (Figure 3H). Significance level p<=0.05. Download Table 3-4, RTF file.

Compression injury showed a decrease in the range of movement during the first 11 d after the injury. From Day 12, a recovery that reached basal values was seen (Fig. 3*D*). As in the irreversible injury, prior to the nerve injury, long and short amplitudes are shown; subsequently, an increase in low amplitudes was observed until Day 11. For Day 12 and up to Day 20, a progressive increase in high amplitudes was observed. However, at no time do they get comparable with the basal state (Fig. 3*E*). When analyzing the frequencies, it was observed that low frequencies predominate during the first 10 d after the injury (0–5 Hz). From Day 11, a variation between 0 and 45 Hz is observed (Fig. 3*F*). Thus, despite an apparent recovery in frequency and range of motion, there was difficulty in making wide movements of the whisker.

Finally, the sham group never lost the range of motion after surgery (Fig. 3*G*). In addition, it showed balanced variations in long and short amplitudes, and at no time did it show a similar behavior to the facial paralysis models (Fig. 3*H*). There is a greater variation in frequency (0–50 Hz) in the movement throughout the assessment sessions (Fig. 3*I*). This indicates that facial paralysis affects the range and frequency of whisker movement. For irreversible paralysis, the effects were permanent, and in reversible paralysis, recovery was partial, but the movement was sufficient to be compared with preinjury states.

### Effect of facial paralysis on facial movement in mice

To assess changes in the faces of mice, a facial movement was tracked by video recording over 2 min and was fragmented into frames. The difference between the first and subsequent frames was calculated by subtracting them (see Materials and Methods). A larger difference means that the mouse is making facial movements, whereas a smaller difference means that there is no movement. During baseline, movement can be observed in the ear, eye area, and whisker area (Extended Data Fig. 4-1*A*). For the transection injury group, movement of the ear can be observed during the 24 h following facial paralysis. However, movement in the eye and whisker area was completely lost. By Days 10 and 20, loss occurred throughout the face (Extended Data Fig. 4-1*B*). For the compression injury group, a loss of movement, like the transection group, was observed on Days 1 and 10. By Day 20, movement can be observed in the eye, whisker, and ear areas (Extended Data Fig. 4-1*B*). Finally, the sham group never lost the ability to move its face during the days evaluated (Extended Data Fig. 4-1*B*). Therefore, facial paralysis by transection and compression affects the ability to move the mouse face. In contrast to previous data, transection had permanent effects, and compression had an apparent recovery.

**Figure 4.**
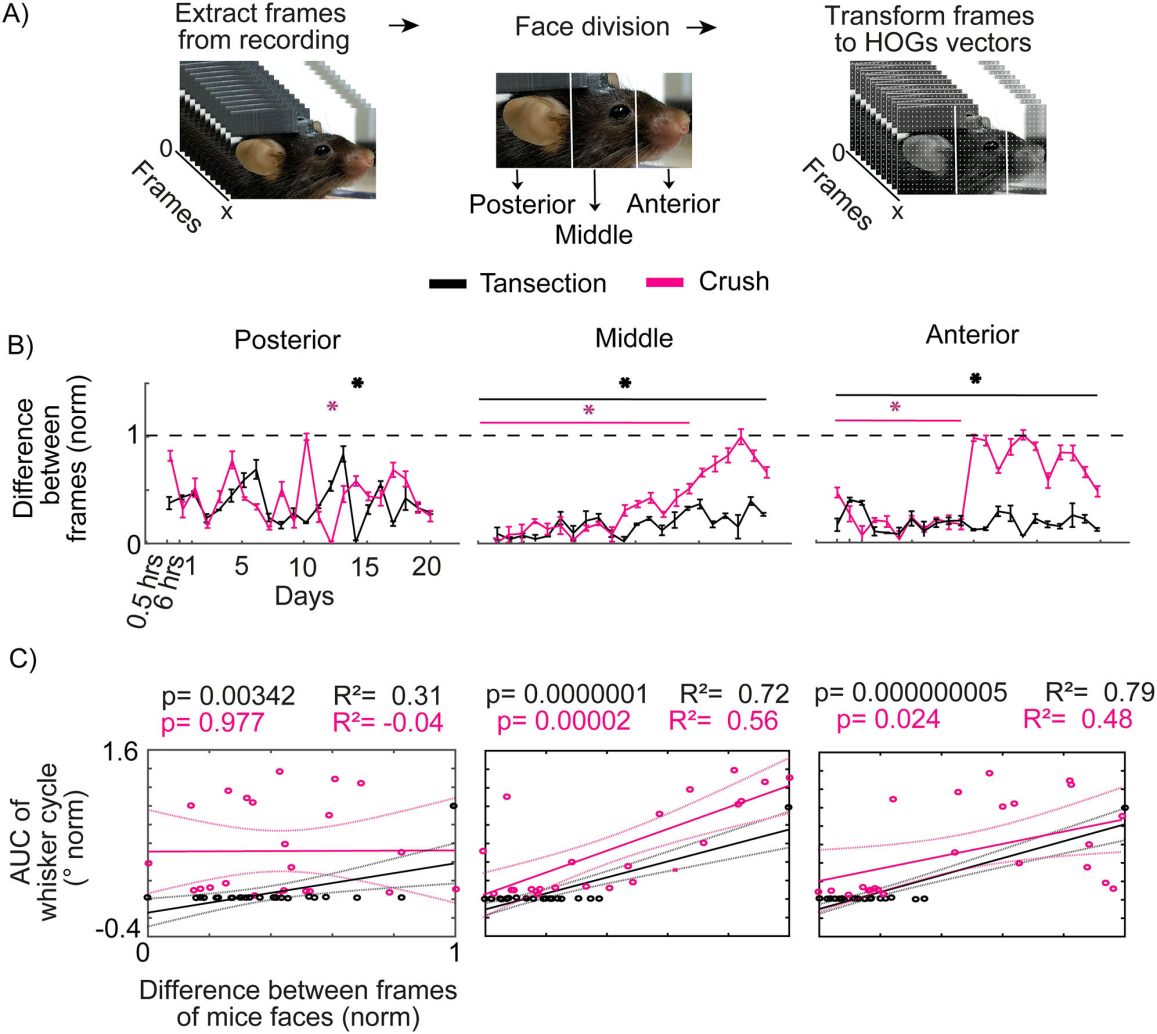
Involvement of the anterior, middle, and front parts of the face in the identification of facial paralysis. ***A***, Schematic of the processing of the video recording of the faces of the mice. ***B***, Differences between HOGs during 2 min of evaluation from half an hour to 20 d after the facial injury (*n* = 3). The dotted line represents the baseline. The left panel symbolizes the posterior area of the face (baseline vs transection *p ≤ 0.05 one-way ANOVA test, Tukey’s test *p* ≤ 0.05; baseline vs crush *p ≤ 0.05 one-way ANOVA test, Tukey’s test *p* ≤ 0.05). The middle panel is the middle area of the face (baseline vs transection *p ≤ 0.05 one-way ANOVA test, Tukey’s test *p* ≤ 0.05; baseline vs crush *p ≤ 0.05 one-way ANOVA test, Tukey’s test *p* ≤ 0.05). The right panel shows the anterior face (baseline vs transection *p ≤ 0.05 one-way ANOVA test, Tukey’s test *p* ≤ 0.05; baseline vs crush *p ≤ 0.05 one-way ANOVA test, Tukey’s test *p* ≤ 0.05). ***C***, Correlation between the difference between HOGs of the face areas of the mice (posterior, left panel; middle, middle panel; and anterior, right panel, *n* = 3) with the area under the curve of the whisker movement cycle. The points represent each of the 23 evaluations performed. The solid line shows the linear regression, and the dotted lines represent the confidence limit. Detailed statistics in Extended Data Tables 4-1–4-2. Complementary information is shown in Extended Data Figures 4-1–4-2 and Table 4-3.

To find out whether facial paralysis affects the mouse's face homogeneously, facial movement analysis by areas was made: anterior (nose, lips, mouth, and whiskers), middle (cheek and eye), and posterior (ear). The frames of each video recording were obtained (*n* = 3,600) and transformed into mathematical vectors (HOGs; Fig. 4*A*). The difference between HOGs was obtained (the subtraction between the first frame minus the subsequent ones). A greater difference indicates that the HOGs changed between frames, which translates into the facial movement of the analyzed area. In the posterior area, for the three groups (transection injury, compression, and sham), a total loss of movement was not observed during all the days of evaluation, and it was comparable to the day before facial injury (Fig. 4*B*; Extended Data Fig. 4-2*A*). In the middle and anterior areas, for the transection injury group, a loss of movement was observed during the 20 d evaluated. For the compression injury group, in the middle area, there was a loss of movement in the first 8 d. Subsequently, an increase in the capacity of movement was observed until Day 15, when the movement was comparable to baseline values. In the anterior area, a loss of movement was observed until Day 9. From Day 10, movement recovered to baseline values (Fig. 4*B*). In the sham group, for the middle and anterior areas, there were some days with a decrease in the movement capacity. Still, these are not significant with respect to the basal movement (Extended Data Fig. 4-2*A*). This shows that facial paralysis affects the movement of the anterior and middle areas of the face, with the eye, cheek, nose, lips, mouth, and mustache being the affected structures.

10.1523/ENEURO.0384-24.2025.f4-1 Figure 4-1 **Facial movement between frames during facial video recording of mice.** A) Heat map of a representative mouse before and B) after facial nerve injury (days 1, 10, and 20); a representative mouse for the transection group in the upper panels; crush in the middle panels; and sham in the lower panels. Download Figure 4-1, TIF file.

10.1523/ENEURO.0384-24.2025.f4-2 Figure 4-2 **Assessment of mouse facial areas in the sham group.** A) Differences between HOGs during 2 minutes of assessment (n = 3), from half an hour to 20 days after a facial injury. The dotted line represents the baseline. The left panel symbolizes the posterior area of ​​the face (Baseline vs Sham p > 0.05 one-way ANOVA test). The middle panel is the middle area of ​​the face (Baseline vs Sham p > 0.05 one-way ANOVA test). The right panel is the anterior area of ​​the face (Baseline vs Sham p > 0.05 one-way ANOVA test). B) Correlation between the difference between the HOGs of the facial areas of the mice (posterior, left panel; middle, middle panel and anterior, right panel, n = 3) and the area under the whisker movement cycle curve. The dots represent each of the 23 assessments performed. The solid line shows the linear regression, and the dotted lines represent the confidence limit. Detailed statistics in Extended Data Table 4-3. Download Figure 4-2, TIF file.

10.1523/ENEURO.0384-24.2025.t4-1 Table 4-1 **Statistical details in the differences between frames in the transection group.** Difference between the first frame with the others in the video, comparison between baseline vs days post facial paralysis (Figure 4B). Significance level p<=0.05. Download Table 4-1, RTF file.

10.1523/ENEURO.0384-24.2025.t4-2 Table 4-2 **Statistical details in the differences between frames in the crush group.** Difference between the first frame with the others in the video, comparison between baseline vs days post facial paralysis (Figure 4B). Significance level p<=0.05. Download Table 4-2, RTF file.

10.1523/ENEURO.0384-24.2025.t4-3 Table 4-3 **Statistical details in the differences between frames in the sham group.** Difference between the first frame with the others in the video, comparison between baseline vs days post facial paralysis (Figure 4-2A). Significance level p<=0.05. Download Table 4-3, RTF file.

### The similarity between the evaluation of facial paralysis by means of whisker movement and the face of mice

Since facial paralysis affects the movements of the mouse's face, the next thing we needed to know was whether the evolution of paralysis is similar in the face as in the mouse's whisker movement. To do this, we sought the dependence between the loss of whisker movement (range of movement determined by the area under the curve of the whisker movement cycle) and the movement of the face areas (difference in HOGs between frames). In the case of the ear area, for the group where the facial nerve was cut (transection group), a positive linear relationship is observed between the data. The opposite is true for the group where the facial nerve was compressed (compression group), where the linear relationship is negative and not significant. This contrasts with the previous data, where it is observed that facial paralysis does not generate a total loss of movement of the ear area (Fig. 4*C*). For the middle and anterior areas in both the compression and transection groups, a linearly positive relationship with the whisker movement range was observed (Fig. 4*C*). For the group that underwent sham surgery (sham group), the relationships of the anterior, middle, and posterior areas with the whisker movement range are linearly positive (Extended Data Fig. 4-2*B*). With this, we show that the movement of the face of mice has a positive relationship with the whisker movement, and, in the presence of facial paralysis (reversible or irreversible), the face shows a loss of movement like that of the whiskers.

### An unbiased inference of experimental facial paralysis using facial movement in mice

**Figure 5.**
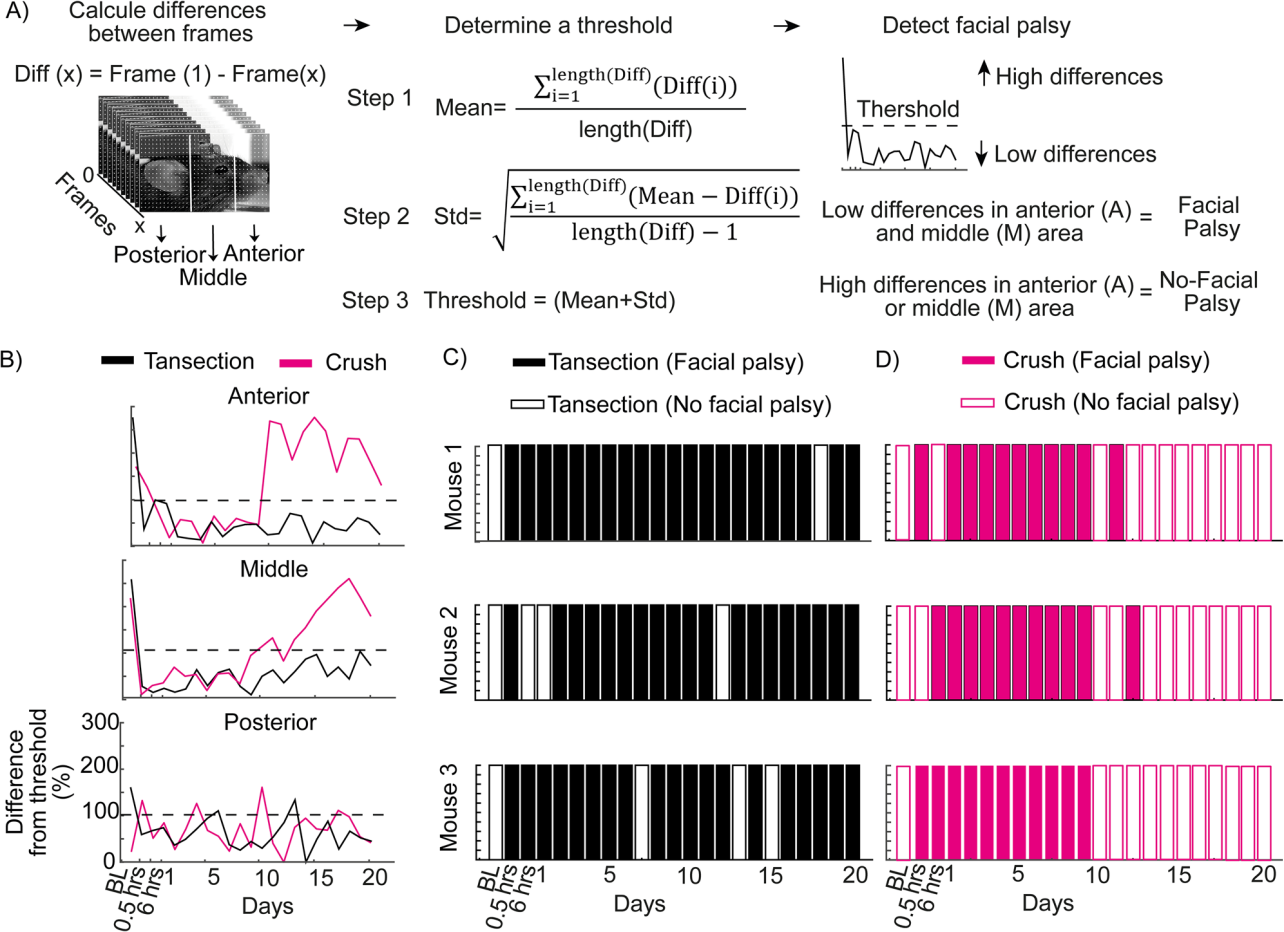
Design and implementation of the FaPA. ***A***, Step-by-step diagram of the FaPA design. ***B***, Percentage of change between the difference of the frames during 2 min of evaluation and the threshold obtained. Use of the threshold for facial paralysis prediction in a binary decision-making system (facial paralysis exists or not) (***C***) for the transection injury group (*n* = 3) and (***D***) for the compression injury group (*n* = 3). Complementary information is shown in Extended Data Figures 5-1–5-2.

One of the advantages of facial paralysis is that we can detect it with the naked eye, and we can observe the moment when an apparent recovery is reached. In this sense, we now have tools that are much more precise than the human eye, such as automated image processing and computerized decision-making, which allow us to predict specific phenomena and classify them. Therefore, we seek to implement these tools to determine the presence of experimental facial paralysis. The first step was to determine a threshold for the movement of the areas of the mouse face, calculated as the average of the difference in HOGs between frames throughout the days of evaluation plus the standard deviation (Fig. 5*A*). This threshold considers the range of movement of all the sessions evaluated.

10.1523/ENEURO.0384-24.2025.f5-1 Figure 5-1 **Step-by-step calculation of FaPA**. Steps 1 to 14 for creating the threshold to generate the FaPA algorithm. From processing the base video (steps 1-4), processing the video with the mouse froze (steps 1-11), calculating the threshold (steps 12-13), and using the algorithm (step 14). Download Figure 5-1, TIF file.

10.1523/ENEURO.0384-24.2025.f5-2 Figure 5-2 **Facial paralysis algorithm efficiency.** A) The left panel shows the level of efficiency of the different algorithms designed to detect facial paralysis in continuous and random data. The middle panel shows the application of each algorithm to infer facial paralysis in a characteristic mouse. The right panel shows the percentage of correct decisions made for each algorithm. The rectangle indicates the designed algorithm with the highest efficiency in predicting paralysis. B) The upper panel shows the efficiency of the algorithm using different amounts of post-facial injury assessments averaged together; each bar indicates the number of assessments used. The low panel shows the percentage of correct decisions made from each algorithm. C) The upper panel shows the efficiency of the algorithm when averaging the baseline with one post-facial injury assessment; each bar indicates the assessment used with the baseline. The low panel shows the percentage of correct decisions made from each algorithm. Download Figure 5-2, TIF file.

Furthermore, it was determined how different the movement of the mouse face is with respect to the threshold. For the transection group in the middle and anterior areas, it can be observed below the threshold throughout the sessions evaluated (Fig. 5*B*). In the posterior area, the motion range did not seem to remain below the threshold. For the compression injury group, in the middle area, the range of motion was below the threshold until Day 13, and in the anterior area, until Day 9. Afterward, the data exceeded the threshold, indicating a recovery of movement in the face of the mice (Fig. 5*B*).

To conclude that a mouse had facial paralysis, a computational decision-making model was used, which considers whether the movement in the areas of the face is below (with paralysis) or above (without paralysis) the threshold (Fig. 5*B*; see Materials and Methods), which we call CA. The efficiency of the CA was demonstrated by making variations in the parameters it uses, which are the areas of the face (summed or independently), data averaged by group or individually, and normalized or non-normalized data (see Materials and Methods). The most efficient CA is the one that considers the sum of the data in the anterior and middle areas, normalized and averaged by group. One consideration for the use of this CA is the high percentage of correct decisions, which occurs when the CA predicts facial paralysis on days when the mouse is paralyzed, considering the detection by whisker movement (Extended Data Fig. 5-1*A*; see Materials and Methods for more details). In this way, the CA detects facial paralysis throughout the days evaluated in mice with transection injury. For compression injuries, the system predicts facial paralysis within the first 12 d. Subsequently, movement is detected in the basal state (Fig. 5*D*).

**Figure 6.**
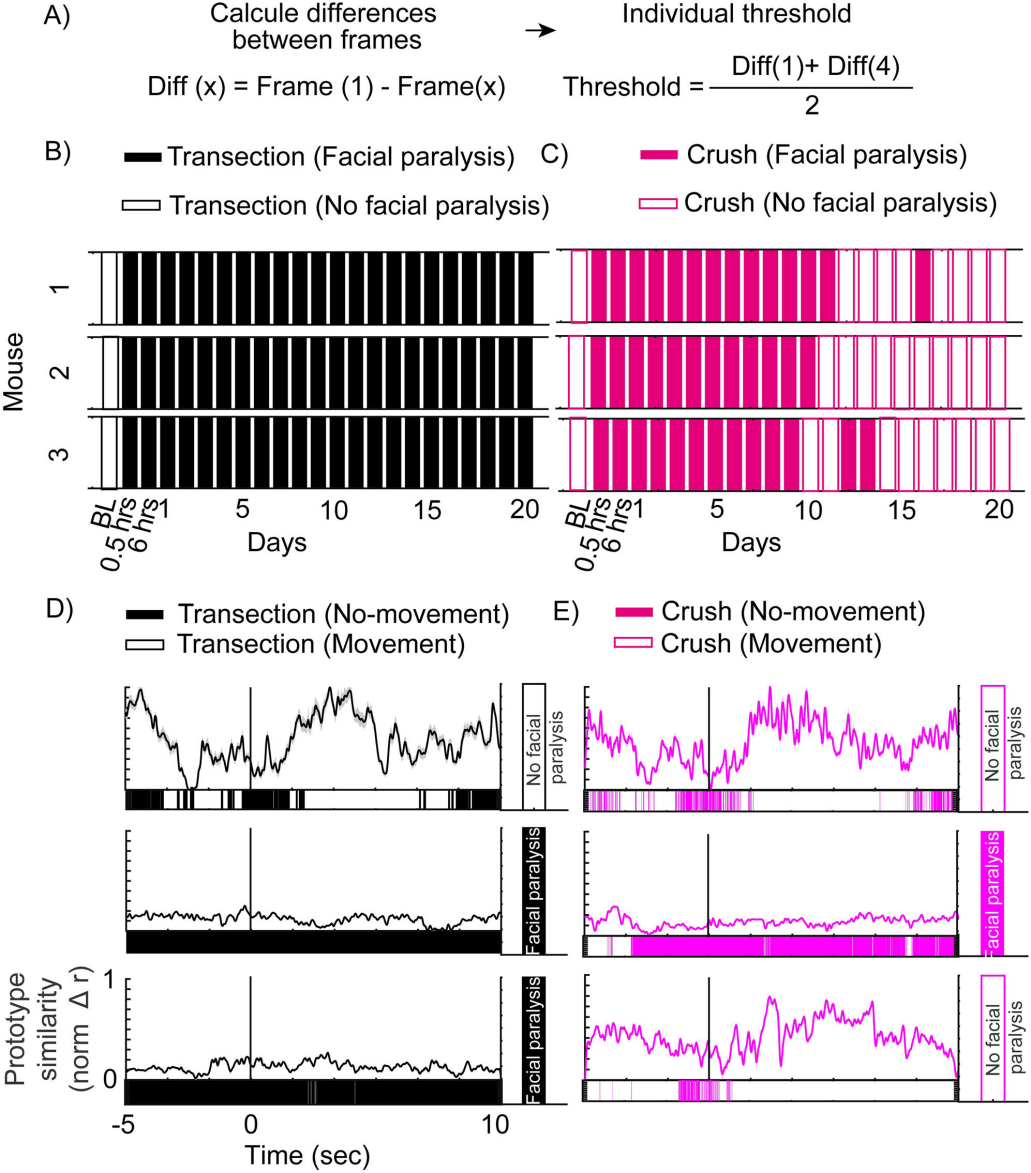
Use of the prediction algorithm during the study of facial expression**. *A***, Design of the algorithm to detect facial paralysis. Detection of facial paralysis using the CA for the transection (***B***, *n* = 3) and compression (***C***, *n* = 3) groups. Tracking the similarity between facial movement and the prototype for the transection (***D***, *n* = 1) and compression (***E***, *n* = 1) groups. The top panels correspond to the baseline evaluation, the middle ones to Day 1, and the bottom ones to Day 20 after facial injury. The bar under each graph indicates the moment in which there is or no movement. The bar on the right shows the conclusion of the CA. The black line at 0 shows the moment of sucrose release. Detailed statistics in Extended Data Table 6-5. Complementary information is shown in Extended Data Figures 6-1–6-2 and Tables 6-1–6-4.

However, the AC still presents some drawbacks since the transection injury group shows days in which the system does not predict the presence of facial paralysis (Fig. 5*C*). Therefore, the decision was made to follow up with the mice individually to find out if the facial information of each subject was sufficient to determine the presence of facial paralysis. In this sense, the number of sessions required to obtain an optimal threshold was first determined. For this purpose, the difference between the HOGs of each frame of the 23 sessions evaluated was used. These differences were averaged and used as a threshold point; 22 different thresholds were created, and in each one, a smaller number of sessions were used on average. In this way, it was observed that two sessions (a baseline evaluation and a paralyzed one) are sufficient to efficiently infer the presence of facial paralysis (Extended Data Fig. 5-1*B*). Subsequently, it was sought which session (of the 22 after facial nerve injury) was the best to create the threshold, where it is observed that no matter which session is used, the CA has the same level of efficiency (Extended Data Fig. 5-1*C*). Therefore, the decision was made to use the session 24 h after the nerve injury; the average of the movement of the anterior and middle zones was taken as a threshold (Fig. 6*A*). In this way, the CA predicts facial paralysis in all mice in the days after the transection injury (Fig. 6*B*). In the compression group, facial paralysis was inference in the first 11 d; in the case of mice one and three, there were some days in which the system detected paralysis after Day 11. However, on Day 17 and up to Day 20, the CA no longer predicts paralysis (Fig. 6*C*). In this way, each mouse can use its facial information to track paralysis. It is capable of being used in the reversible and irreversible models, and it is not necessary to use stimuli that provoke facial movement.

10.1523/ENEURO.0384-24.2025.f6-1 Figure 6-1 Facial paralysis detection between fixed head restraint systems and between sexes. A) Area under the curve of the whisker movement cycle over 20 years of transection and compression paralysis in two different fixed head restraint systems (metal bar and bar made of PLA). The dotted line indicates the baseline value (n = 3 per group). B) Use of the FaPA for the detection of facial paralysis in a mouse characteristic of the compression and transection group of each fixation system. C) Percentage of correct decisions made by the FaPA for the transection and compression group (n = 3 per group). D) Area under the curve of the whisker movement cycle over 20 years of transection and compression paralysis between males and females (n = 3 per group). The dotted line indicates the baseline value. E) Use of the FaPA for the detection of facial paralysis in a mouse characteristic of the compression and transection group in males and females. C) Percentage of correct decisions made by FaPA for the transection and compression group (n = 3 per group). Detailed statistics in Extended Data Table 6-1, Table 6-2, and Table 6-3. Download Figure 6-1, TIF file.

10.1523/ENEURO.0384-24.2025.f6-2 Figure 6-2 **Gustatory stimuli induce facial expressions.** A) Schematic of the analysis process of the video recordings to detect facial expressions. B) Heat map of the correlation of the frames of 3 stimuli (n = 210 in one mouse) before and after the release of sucrose, water, and quinine, sorted by clusters. C) Grouping by clusters of the frames after oral stimulation with sucrose, water, and quinine. D) Schematic of the creation of pleasant, unpleasant, and neutral prototypes. E) Similarity of the prototypes with the moments of oral stimulation with the different solutions (sucrose, quinine and water) in three mice. The dotted line at 0 shows the moment of the release of solutions. Detailed statistics in Extended Data Table 6-4. Download Figure 6-2, TIF file.

10.1523/ENEURO.0384-24.2025.t6-1 Table 6-1 **Statistical details in whisker movement with facial paralysis in two different bars and sex.** Difference in whisker movements between bars and sex in transection and crush models (Figure 6-1A and Figure 6-1D). Significance level p<=0.05. Download Table 6-1, RTF file.

10.1523/ENEURO.0384-24.2025.t6-2 Table 6-2 **Statistical details in whisker movement with facial paralysis between systems.** Difference in area under the curve between baseline vs days post facial paralysis in transection, and crush groups. MBT = metallic bar in the transection group, PBT = PLA bar in the transection group, MBC = metallic bar in the crush group, and PBC = PLA bar in the crush group (Figure 6-1A). Significance level p<=0.05. Download Table 6-2, RTF file.

10.1523/ENEURO.0384-24.2025.t6-3 Table 6-3 **Statistical details in whisker movement with facial paralysis between sex.** Difference in area under the curve between baseline vs days post facial paralysis in transection, and crush groups. MT = male in transection group, MC = male in crush group, FT = female in transection group and FC = female in crush group (Figure 6-1D). Significance level p<=0.05. Download Table 6-3, RTF file.

10.1523/ENEURO.0384-24.2025.t6-4 Table 6-4 **Statistical details in facial expression before and after oral stimulation with solutions.** Comparison between 10 seconds pre- and post-stimulation with sucrose, quinine, or water using the pleasure, disgust, and neutral prototype (Figure 6-2E). Significance level p<=0.05. Download Table 6-4, RTF file.

10.1523/ENEURO.0384-24.2025.t6-5 Table 6-5 **Statistical details in facial expression between baseline vs facial paralysis.** The similarity of pleasure prototype for 10 seconds after oral stimulation with sucrose between baseline, day 1, and day 20 post facial paralysis in transection and crush group (Figure 6D and Figure 6E). Significance level p<=0.05. Download Table 6-5, RTF file.

For the final validation of the algorithm, two different fixation bars were used (one metallic and one made of PLA). The test was carried out on three mice with transection and compression in each of the systems. No difference in whisker movement was found between the systems for both the compression and transection models (Extended Data Fig. 6-1*A*). Similarly, the algorithm can identify reversible and irreversible facial paralysis in the two restraint systems accurately (Extended Data Fig. 6-1*B,C*).

Finally, no difference was found in the transection and compression models when comparing males and females. When analyzing males and females daily in the transection model, we did not find differences between them, which tells us that irreversible facial paralysis is the same regardless of sex. However, in the compression model, faster recovery is observed in females, occurring during the first 8 d after injury, and in the case of males, it occurs on Day 10 (Extended Data Fig. 6-1*D*). In this sense, the algorithm can infer reversible and irreversible facial paralysis in males and females accurately. It can predict this difference in recovery time between males and females in the reversible facial paralysis model (Extended Data Fig. 6-1*E,F*).

### Analysis of gustatory facial movements impaired by facial nerve injury

To understand facial paralysis in depth, it is necessary to study how affected the function of facial movement is and the muscles involved. Therefore, one of the goals to be achieved is to be able to predict facial paralysis with the CA that is developed during behavioral protocols. To do this, facial paralysis was inferred while performing a protocol of consumption of solutions for the study of facial expressions adapted from the protocol developed by Dolensek et al., (2020). We released five drops of 4 μl each of different solutions (bitter, quinine; sweet, sucrose; and neutral, water) to produce a facial movement associated with an emotional component (quinine, unpleasant; sucrose, pleasant; and water, neutral). Such movements were video-recorded, fragmented into frames, and transformed into mathematical vectors (HOGs; Extended Data Fig. 6-2*A*).

To study facial movements associated with the consumption of a solution, the change in the face of the mice before and after stimulation was determined. For this, frames from three stimuli (*n* = 210) were used, which were organized and grouped into clusters using a hierarchical tree analysis (Extended Data Fig. 6-2*B*). It can be observed that for each substance (sucrose, quinine, and water), the frames before and after stimulation are grouped into two different clusters. This indicates that the face of the mouse changes when it is orally stimulated with respect to a basal state (Extended Data Fig. 6-2*B*). Once we knew that the face changed during stimulation, we looked for the difference between the movement patterns of each administered solution. To do this, a cluster grouping was made (using a stochastic neighbor analysis of t-distribution) between frames of each solution (*n* = 120 per solution). It can be observed that each solution has its cluster (Extended Data Fig. 6-2*C*), which indicates that facial movement follows a different pattern for each solution, and this is related to the different facial movements evoked by oral stimulation (Dolensek et al., 2020).

Regarding the above, the characteristic facial movement patterns of each solution can be extracted to generate a movement prototype (see Materials and Methods; Extended Data Fig. 6-2*D*). Each prototype was associated with an emotion (pleasure, sucrose; disgust, quinine; and neutral, water) and compared with each stimulus. It can be observed that stimulation with sucrose has a high similarity with the pleasant prototype but not with the neutral and disgust prototypes. Something similar occurs for quinine stimulation, which has a high similarity with the disgust prototype and not with the other two. Water follows the same pattern, where it only increases the similarity with the neutral prototype (Extended Data Fig. 6-2*E*). This shows us that facial movement is prototypical and is related to the solution administered and provokes a particular emotion.

After that, the developed CA was used to determine the presence of facial paralysis at the time of stimulation with sucrose. The system was compared with each frame during oral stimulation (*n* = 450). In this way, we have two moments during the session: with movement and without movement. The percentage of each of the moments is obtained, and the system makes its conclusion, whether there is paralysis (>90% of frames without movement) or not. A mouse with compression and transection injury was monitored, which, prior to nerve injury, we can observe a high similarity to the pleasure prototype after oral stimulation. In addition, during the increase in similarity, the system detects that there is facial movement. With this, the system concludes that there is no facial paralysis (Fig. 6*D,E*). For the mouse with transection injury, we can observe on Days 1 and 20 after surgery that there is no facial movement, and neither does the similarity with the pleasant prototype increase, so the system concludes that there is facial paralysis on both days (Fig. 6*D*). For the mouse with compression injury, 24 h after surgery, a loss of movement is observed, concluding that there is facial paralysis. However, by Day 20, the movement recovers and is like the pleasant prototype, which results in there being no facial paralysis. In this way, the developed AC allows us to study different behaviors associated with facial movement and efficiently detects the presence of facial paralysis in the different models used.

### Facial paralysis inference during electrophysiological assessment in the ALM

**Figure 7.**
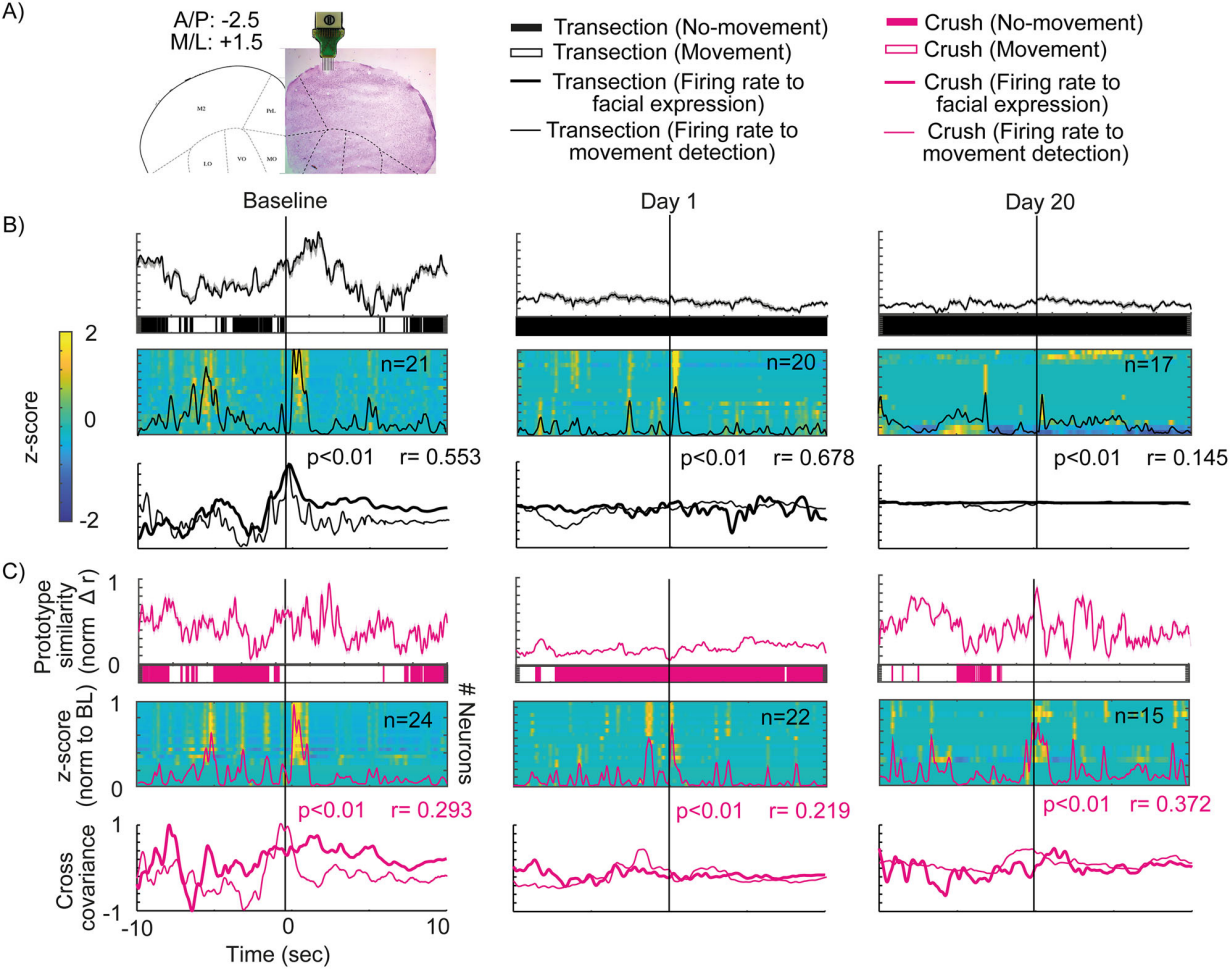
Electrophysiological recordings in the ALM during facial expression and movement. ***A***, Location of the electrode array implanted in the ALM in the histological section of a recorded mouse. ***B***, ***C***, The top panel depicts the tracking of the similarity of the facial expression with the pleasurable prototype. In the top panel, the bar below shows the moment of facial movement. The middle panel shows the heat map of the population neuronal activity in the ALM; the line inside the heat map is the population average. The bottom panel shows the cross-covariance of neuronal activity with facial expression (thick line) and movement (thin line); (***B***) for the transection lesion group (*n* = 2) and (***C***) for compression (*n* = 2). The line aligned at 0 shows the moment of facial expression onset. Detailed statistics in Extended Data Table 7-1. Complementary information is shown in Extended Data Table 7-2.

As a final goal, we proposed to use the designed AC during electrophysiological recording in the ALM while the previously mentioned facial expression assessment protocol was being carried out (Fig. 7*A*). Neurons associated with facial expression and movement were used. During the basal state, we can observe an increase in the ALM neuronal activity at the moment that the similarity with the pleasurable prototype increases, and the algorithm detects that there is movement in the transection and compression lesion groups (Fig. 7*B,C*). Finally, the cross-covariance analysis between the firing frequency and the similarity of the facial movement with the pleasurable prototype shows that the neuronal activity occurs when the facial expression begins and is maintained during the first seconds in which it occurs. With the same analysis, comparing the firing frequency with the moments of facial movement, it is shown that neuronal activity occurs during facial movement (Fig. 7*B,C*). The cross-covariance of neuronal activity with facial expression and movement shows a significant positive correlation (Fig. 7*B,C*).

10.1523/ENEURO.0384-24.2025.f7-1 Figure 7-1 **Electrophysiological recordings in ALM during walking and activation of the solution infusion pump.** The upper panel shows the heat map of population activity in ALM associated with the activation of the solution infusion pump, and the lower panel shows mouse walking for A) the transection group (n = 2) and B) compression (n = 2). The black line aligned at 0 indicates the moment when the pump is activated, and walking begins. Detailed statistics in Extended Data Table 7-2. Download Figure 7-1, TIF file.

10.1523/ENEURO.0384-24.2025.t7-1 Table 7-1 **Statistical details in population neuronal activity in ALM pre- and post-facial expression.** Differences between z-score pre- and post-oral stimulation with sucrose in population neuronal activity of two mice (Figure 7B and Figure 7C). Significance level p<=0.05. Download Table 7-1, RTF file.

10.1523/ENEURO.0384-24.2025.t7-2 Table 7-2 **Statistical details in population neuronal activity in ALM pre- and post-control events.** Differences between z-score pre- and post-walking and activation of a stepper motor in population neuronal activity of two mice (Figure 7-1A and Figure 7-1B). Significance level p<=0.05. Download Table 7-2, RTF file.

For the transection injury group, 24 h after the injury, facial expression and movement do not exist; however, an increase in the firing frequency after oral stimulation can still be seen, but it is not comparable to baseline values (Fig. 7*B*). By Day 20, facial movement does not occur, and the firing frequency decreases further compared with Day 1 after the injury (Fig. 7*B*). In this sense, the cross-covariance between the firing frequency and facial expression does not show a correlation; the same occurs between the firing frequency and the moment of movement. Likewise, both cross-covariance values show a high, significantly positive correlation (Fig. 7*B*). This shows us that neuronal activity in the ALM changes as facial paralysis progresses, and it does not occur in the same way as the loss of facial movement, which is immediate with respect to the injury.

In the compression group, 24 h after the injury, something similar occurs as with the transection group. The firing frequency decreases with respect to basal values; there is no similarity with the pleasurable prototype, and there is no facial movement (Fig. 7*C*). By Day 20, there is an increase in the similarity to the pleasurable prototype and facial movement; likewise, the firing frequency increases to values like the basal state. The cross-covariance between facial expression and firing frequency shows that neuronal activity occurs after the facial expression begins. In the case of the comparison with the moment of movement, neuronal activity occurs during facial movement (Fig. 7*C*). With this, it is shown that neuronal activity is associated with the capacity for facial movement.

To confirm that the neurons analyzed only respond to facial movement, the neuronal activity was aligned with the walking movement. It can be observed that the neurons do not show an increase in their firing frequency when initiating and maintaining walking (Extended Data Fig. 7-1*A,B*). Subsequently, the same activity was aligned with the activation of the infusion pump (see Materials and Methods) without administering a solution; similar to our previous result, the firing frequency has no significant modulation (Extended Data Fig. 7-1*A,B*). This demonstrates that the neuronal activity in the transection and compression groups is associated with a facial response that correlates to oral stimulation.

## Discussion

In this article, we propose for the first time a model for studying facial paralysis in the mouse, which allows for easy, rapid, and automated assessment of nerve injury progression. FaPA is a tool that allows us to understand the progression of experimental facial paralysis in mice, which is sensitive to this type of injury to the facial nerve, reflects changes in whisker movement, is sensitive to changes in the stereotyped movement of facial expression, and is a tool that correlates changes in neuronal activity in the premotor cortex of the rodent.

Facial nerve compression and transection techniques in murine models have been the most widely used to model facial paralysis (Chacon et al., 2020) due to their similarity with the clinical prognosis. The rapid recovery that we can observe in the compression model is comparable to that of most patients with Bell's palsy (Holland and Weiner, 2004). In contrast, the lack of recovery in transection resembles cases of poor prognosis of facial paralysis caused by herpes zoster or trauma (Holland and Weiner, 2004). However, these models have limitations since they do not capture all varieties in facial paralysis and exclude etiologies such as tumors, otitis media, or neonatal conditions. These techniques allow us to have reversible (compression) and irreversible (transection) models capable of comparing and evaluating facial paralysis for the first time in mice.

The results of our study in mice corroborate previous findings in Wistar rats, showing that compression induces a rapid recovery in whisker movement amplitude. In contrast, transection shows no recovery over time (Hadlock et al., 2010).

In addition to assessing whisker amplitude, our study explored changes in whisker movement frequency. Jowett demonstrated that after nerve injury, rats showed a decrease in whisker spectral power (Attiah et al., 2017). Our study assessed frequency for 20 d in both models, identifying the prevalence of low frequencies (0–5 Hz) after the injury. The absence of a specific stimulus to induce whisker movement limits comparability. However, our findings support the hypothesis that facial nerve injury affects not only whisker movement amplitude but also frequency.

This work proposes the study of facial paralysis using facial expression. For this purpose, we sought to use video recordings of the faces of mice without any stimulus and without the need to mark or cut the whiskers (Severson et al., 2019; Vajtay et al., 2019). In addition, the importance of considering the complete movement of the face in the study of facial movement and conditions such as facial paralysis has been shown (Severson et al., 2019). Our results show muscle function loss in two of the three facial regions studied (the middle and anterior areas). This might be related to the impact of the facial injury on the musculature of the mouse ear and its need for sound sensation (Cakmak, 2019; Clayton et al., 2024). The effect of facial paralysis on whisker movement is related to the mechanical technique used to produce the injury (Hadlock et al., 2010).

In contrast, the results show a temporal loss of facial movement function, like the loss of whisker movement. They are also dependent on the type of injury to the facial nerve. This is observed in the anterior and middle areas of the face.

Currently, the use of digital tools and artificial intelligence for the study of the complexity of facial movement has been shown more frequently (Tanaka et al., 2023; Syeda et al., 2024). This work proposes a bimodal detection algorithm to infer facial paralysis automatically. It obtains information on the movement of the mouse's face in three segments since its structures are highly involved in facial movement (Le Moene and Larsson, 2023; Tanaka et al., 2023). The results show us the importance of using the middle and anterior areas of the mouse's face for the automated detection of facial paralysis, despite the fact that the ear area is essential for generating a facial expression (Dolensek et al., 2020).

Furthermore, it is shown that the information specific to each mouse is sufficient to feed the algorithm and monitor each subject's facial paralysis. This can help us understand whether this condition has unique effects on each mouse or can be studied further as a group.

In this study, we propose using artificial vision to characterize the effects of facial nerve injury on musculature movements. For this purpose, we used the facial response elicited by a sucrose solution, as it has been shown that the facial changes are proportional to the intensity of the hedonic stimuli (Le Moene and Larsson, 2023) and remain consistent when evaluated in a head-fixed model through video recording analysis, allowing the determination of the internal state of mice in a quantitative manner (W. R. Li et al., 2023). As such, the reduced motion observed after the facial nerve injury is related to the inability to perform the facial movements rather than a devaluation of the reward being delivered.

To corroborate the usefulness of this tool in assessing facial paralysis in mice, we analyzed its neural correlates in the ALM, a possible homolog of primates’ premotor cortex. Our data confirm that spontaneous orofacial responses are initiated and executed in this cortex (Giordano et al., 2023) and that the neural modulation associated with orofacial movement disappears under conditions of facial paralysis.

On the contrary, our data also suggest that neuronal modulation of the premotor cortex is reestablished on Day 20 after nerve injury in the reversible model. Therefore, the experimental model works to see fine changes at the cortical level associated with the natural history of experimental facial paralysis.

Its proposal could be further improved by incorporating deep-learning strategies for online analysis, which would be fascinating to implement. Applying a similar analysis to freely moving animals would be more engaging and clinically relevant; we used a fixed-head mouse model, which restricts the evaluation time and imposes limitations on analyzing facial movement. With freely moving mice, it would be more feasible to avoid altering the physiology of facial movement, though this would require additional work in data processing.

Further in-depth experiments are still required to analyze aspects of planning and execution, also attributed to the ALM. (N. Li et al., 2015), and that in other experimental conditions gives us the perspective of treating experimental facial paralysis in rodents with neuroprostheses or specific neuromodulation.

## Synthesis

Reviewing Editor: Niraj Desai, National Institute of Neurological Disorders and Stroke Decisions are customarily a result of the Reviewing Editor and the peer reviewers coming together and discussing their recommendations until a consensus is reached. When revisions are invited, a fact-based synthesis statement explaining their decision and outlining what is needed to prepare a revision will be listed below. The following reviewer(s) agreed to reveal their identity: Atika Syeda.

Two reviewers and I have read your manuscript and find much to admire. We believe your study could be a valuable contribution to the body of work eNeuro has published. Both reviewers recommended some changes that we all (all three of us) believe could improve the manuscript considerably. Ideally, you'll be able to respond to these comments within two months' time; but if longer is necessary, so be it.

This is a good study and I very much hope you revise and resubmit it to eNeuro.

REVIEWER 1

The manuscript proposes an algorithm for facial paralysis detection in mice. The proposed algorithm relies on a decision model selected from 9 models. However, the explanation of the methods used for evaluation and information about the results are insufficient to evaluate whether the algorithm would work on a different dataset. The paper proposes the algorithm as a tool that other labs can use but the manuscript and associated code does not have enough information on how the users can employ the tool. I propose a few analyses and questions below:

1. Can the authors explain the choice of 9 decision models? Please provide more details on how the model was selected i.e. for evaluation results in Figure 5-1 and more details in methods. Please add information on whether train and test data were used and show results for both.

2. The analysis on the videos was performed on a 2-minute window for the different conditions. Please explain how the 2-minute window was selected and whether single trial was used for different conditions (baseline, day 0...) or multiple trials?

3. It is unclear from the manuscript how the emotional prototype was selected. The approach seems similar to Dolensek et al 2020 and if so, please cite the paper. Also, please include how many mice were used for the prototype? Is there a train and test dataset used for testing generalization of the emotional prototype?

4. What is the reasoning for dividing facial movements into the three sections (middle, anterior, posterior)? Add comparison for whole face/frame differences in Fig. 4. Would the results still hold for full face analysis?

5. Other additional comments and questions for authors:

- a number of spelling errors throughout the paper. Please revise.

- For ALM recordings n=2 mice were used. I would suggest power analysis.

- Does the whisker painting interfe with facial video recordings and movement of the face?

- Discuss limitations: can it only be used for head-fixed setups? Could a similar approach be used without HOGs but existing methods, such as motion energy or video PCs?

-

REVIEWER 2

In this paper, the authors employed an object detection approach leveraging a histogram of oriented gradients (HOG) feature to analyze facial dynamics in mice subjected to either facial nerve crush or transection. Their findings demonstrated that a facial nerve crush led to a transient reduction in facial movements, while a facial nerve transection resulted in a sustained loss of these movements. By arbitrarily setting a threshold value, the authors defined a state with reduced facial dynamics as 'facial paralysis' in their analysis. This algorithm was named FaPDA by the authors. It seems that the authors feel a need to assert novelty, but the image analysis algorithm employed in this study is quite common in the examination of animal behavior dynamics. The paper needs to be revised to accurately situate the findings within the context of existing literature and should be presented in a scholarly scientific manner.

Major Concerns:

1. As described in the general comment above, overall manuscript should be rewritten more honestly, without hyping the novelty. In particular, the authors should explicitly acknowledge that the image analysis and the mouse model of facial paralysis utilized in this study are pre-existing approaches in the Introduction.

2. The specific research question regarding the studies of facial paralysis in mice is not clear from the current manuscript. In addition, the necessity for automatic detection of facial paralysis in mice is also ambiguous. If the absence of facial movement can be detectable by human eyes, such technology may be unnecessary. These points should be clarified in the Abstract and Introduction.

3. The background of the study should be detailed in the Introduction rather than the Discussion (e.g., lines 611-).

4. The section 'Facial paralysis detection algorithm applied in mice (FaPDA) design' in the Methods is overly complex and difficult to understand. The authors should revise this section more confluently.

5. For HOG feature-based image analysis of facial dynamics, proper alignment of images is crucial to mitigate movement artifacts in animals. The authors should describe how to align the images in the dataset in the Method section.

6. The term 'detection' as used in the title and elsewhere seems exaggerated. The authors merely defined a state with reduced facial dynamics in mice with facial nerve injury as 'facial paralysis' in their analysis, without evidence validating these events as 'true' facial paralysis. Therefore, the authors should use more precise terms such as 'prediction' or 'inference.'

7. The determination of the threshold value is critical for the accuracy of the classifier model. Although the authors used 'Mean + Std' as a threshold in Figure 5, this value should be optimized more carefully.

8. The term 'automatic' used from line 450 is misleading, because the authors conducted the image analyses post hoc. The term 'unbiased' would be more appropriate.

9. The significance of the section 'Detection of facial paralysis during oral stimulation with sucrose' is unclear. This experiment seems to be an adaptation of a previous work by Delensek and colleagues (Science 2020: doi: 10.1126/science.aaz9468.). The authors should revise this section without overstating novelty or consider removing this figure and the accompanying text.

10. The authors are strongly recommended to send the manuscript to professional scientific/language proof-reading services to improve the writing style. Additionally, the authors are encouraged to unify the styles of figures (e.g. font/size of letters, thickness of lines).

Minor Concerns:

1. The mouse strain used in the study is described as 'C57BL/6.' However, phenotypes of C57BL/6 mice can vary significantly depending on their substrain, as noted in the literature (e.g., Mekada and Yoshiki 2021; doi: 10.1538/expanim.20-0158) and The Jackson Laboratory website (https://www.jax.org/news-and-insights/jax-blog/2016/june/there-is-no-such-thing-as-a-b6-mouse). The authors should provide information on the substrain and vendor of the mice.

2. In line 89, the authors stated, 'Twelve male and female C57BL/6 mice were used, aged 2 to 6 months.' The authors should discuss whether the differences in sex and age of the mice could impact the results.

3. Why did the authors apply ~8 kPa to induce facial compression, as described in Figure 1C? Is there a rationale or previous literature supporting this procedure?

4. In lines 42-44, the authors stated, 'However, to apply these strategies, specialized systems are required, as well as specific preparation of study subjects and movement restriction.' Given that the authors' technique also requires specialized devices such as a head-fixation device and treadmill, this statement is incorrect and should be revised.

5. In line 128, the authors wrote, 'Twenty-three measurements,' but it is unclear what was measured 23 times.

6. Typo in Figure 4A center: 'divition.'

## Author Response

Responses to reviewers (in red) Synthesis Statement for Author (Required):

Two reviewers and I have read your manuscript and find much to admire. We believe your study could be a valuable contribution to the body of work eNeuro has published. Both reviewers recommended some changes that we all (all three of us) believe could improve the manuscript considerably. Ideally, you'll be able to respond to these comments within two months' time; but if longer is necessary, so be it.

This is a good study and I very much hope you revise and resubmit it to eNeuro.

We appreciate your taking the time to review and suggest improvements to the article; it improved a lot.

REVIEWER 1 The manuscript proposes an algorithm for facial paralysis detection in mice. The proposed algorithm relies on a decision model selected from 9 models. However, the explanation of the methods used for evaluation and information about the results are insufficient to evaluate whether the algorithm would work on a different dataset. The paper proposes the algorithm as a tool that other labs can use but the manuscript and associated code does not have enough information on how the users can employ the tool. I propose a few analyses and questions below:

Thank you for the review; we addressed your suggestions and comments, which greatly improved the article.

1. Can the authors explain the choice of 9 decision models? Please provide more details on how the model was selected i.e. for evaluation results in Figure 5-1 and more details in methods. Please add information on whether train and test data were used and show results for both.

Thanks for this suggestion. The decision to use a model is made with respect to the system's percentage of correct decisions. This is used to decide which parts of the face to use for the system and the number of sessions necessary. We added the changes in Figure 5-1 (in the figure caption and the methods section).

2. The analysis on the videos was performed on a 2-minute window for the different conditions. Please explain how the 2-minute window was selected and whether single trial was used for different conditions (baseline, day 0...) or multiple trials? We added a better explanation of the facial paralysis model evaluation (page 4).

Two minutes of total recording were used, considering one session; the mice were 2 not subjected to any stimulation; they were only placed in the fixed head system, and the video recording of the face and whiskers of the mice was obtained at the same time. Figure 2-1 analyzes the movement of whiskers during the basal state of the mice, where without any stimulation, the mice move their whiskers, which can discriminate when there is movement or not. No longer experiments were done since the two minutes of recording analyzed were enough to be able to discriminate between these two states (movement and without movement in the whiskers and face of the mice (Figure 2-1 and 4-1)).

3. It is unclear from the manuscript how the emotional prototype was selected. The approach seems similar to Dolensek et al 2020 and if so, please cite the paper.

Also, please include how many mice were used for the prototype? Is there a train and test dataset used for testing generalization of the emotional prototype? The facial prototype was created using a mouse independent of the facial paralysis experiments; the method to obtain it is based on the Dolensek protocol, which was adapted to our task. An intraoral cannula was used instead of a delivery nozzle, and a controlled amount of solution (32 microliters) was released (page 8 in the section on the creation of the facial prototype and oral stimulation test).

The pleasant prototype extracted from this single mouse was applied to the rest of the mice subjected to facial paralysis models.

4. What is the reasoning for dividing facial movements into the three sections (middle, anterior, posterior)? Add comparison for whole face/frame differences in Fig. 4. Would the results still hold for full face analysis? The decision to divide the face was based on the percentage of correct decisions made when creating the decision-making model, considering the entire face (Figure 5-1 A). In this sense, the decision is made to divide the face into three sections by the structures innervated by each branch of the facial nerve. Our data show that the middle and anterior zones follow a loss of movement like the findings in the whisker analysis (Figure 4C). We believe that the greatest contribution and changes occur in the middle and anterior portion, based on the motor innervation of the facial nerve. However, if the entire face can be used, it is not difficult to divide it into the algorithm, and the sensitivity of detection decreases (Figure 5-2). It would be necessary to evaluate in other models of orofacial motor disorders whether ear movement is crucial, for example, in cases of Parkinson's. However, for the facial paralysis model, the greatest sensitivity is found in the anterior evaluation of the rodent's face.

3 5. Other additional comments and questions for authors:

- a number of spelling errors throughout the paper. Please revise.

Thank you. We checked the errors in detail.

- For ALM recordings n=2 mice were used. I would suggest power analysis.

Thanks for the suggestion. We used a small number of mice because we only wanted to represent the changes in the anterolateral motor cortex. Therefore, we do not see the need to increase the number of animals but only to suggest to the reader that the model works for studying subcircuits. We will leave more details for later publications.

- Does the whisker painting interfe with facial video recordings and movement of the face? We used experiments with mice with painted whiskers and others without, no variations were found in the analysis of facial expressions between them.

- Discuss limitations: can it only be used for head-fixed setups? Could a similar approach be used without HOGs but existing methods, such as motion energy or video PCs? So far, it has only been used in fixed-head systems; two different fixed-head systems were used (the variation in the systems was the method of securing the device implanted in the skull of the mice), in which the algorithm can be used to detect paralysis. We have unpublished data in which we have implemented this form of analysis using HOGS in freely moving animals; however, we have not evaluated this model of freely moving mice with facial paralysis.

- REVIEWER 2 In this paper, the authors employed an object detection approach leveraging a histogram of oriented gradients (HOG) feature to analyze facial dynamics in mice subjected to either facial nerve crush or transection. Their findings demonstrated that a facial nerve crush led to a transient reduction in facial movements, while a facial nerve transection resulted in a sustained loss of these movements. By arbitrarily setting a threshold value, the authors defined a state with reduced facial dynamics as 'facial paralysis' in their analysis. This algorithm was named FaPDA by the authors. It seems that the authors feel a need to assert novelty, but the image analysis algorithm employed in this study is quite common in the 4 examination of animal behavior dynamics. The paper needs to be revised to accurately situate the findings within the context of existing literature and should be presented in a scholarly scientific manner.

Thank you very much for taking the time to review and make suggestions. We have addressed all your suggestions, and the article has certainly improved.

Major Concerns:

1. As described in the general comment above, overall manuscript should be rewritten more honestly, without hyping the novelty. In particular, the authors should explicitly acknowledge that the image analysis and the mouse model of facial paralysis utilized in this study are pre-existing approaches in the Introduction.

We added this information to the introduction and changed the article's name to be more honest about the analysis's novelty level. Thanks for the suggestion.

2. The specific research question regarding the studies of facial paralysis in mice is not clear from the current manuscript. In addition, the necessity for automatic detection of facial paralysis in mice is also ambiguous. If the absence of facial movement can be detectable by human eyes, such technology may be unnecessary. These points should be clarified in the Abstract and Introduction.

Thank you for the suggestion. We added the research question in the introduction and more details to the abstract so that the article's contribution is not ambiguous (page 2).

3. The background of the study should be detailed in the Introduction rather than the Discussion (e.g., lines 611-).

We added the information to the introduction (page 2) 4. The section 'Facial paralysis detection algorithm applied in mice (FaPDA) design' in the Methods is overly complex and difficult to understand. The authors should revise this section more confluently.

We have made some changes in this section, and we hope it will be more confluent. We also made improvements to the explanation of the MATLAB script so that it can be used easily.

5. For HOG feature-based image analysis of facial dynamics, proper alignment of 5 images is crucial to mitigate movement artifacts in animals. The authors should describe how to align the images in the dataset in the Method section.

A MATLAB image processing tool was used to align the videos, ensuring that the position of the mouse's face was the same. Thus, the emotional prototype could be compared. It was added to page 5: Facial motion analysis. Shortly, each video was aligned with a baseline video. For the alignment, four anatomical points were considered as references, two in the eye and two in the nose. Using the imagetform function and considering the anatomical points; the video was rotated to the position of the baseline video until they were aligned. In this way, the variation between each video recording was reduced and comparison between them was made possible.

6. The term 'detection' as used in the title and elsewhere seems exaggerated. The authors merely defined a state with reduced facial dynamics in mice with facial nerve injury as 'facial paralysis' in their analysis, without evidence validating these events as 'true' facial paralysis. Therefore, the authors should use more precise terms such as 'prediction' or 'inference.' Thanks for the suggestion. We made the suggested changes in the title and body of the manuscript.

7. The determination of the threshold value is critical for the accuracy of the classifier model. Although the authors used 'Mean + Std' as a threshold in Figure 5, this value should be optimized more carefully.

The average plus the standard deviation of the variation between each frame of the videos obtained (the baseline and post-surgery evaluation) is used. This allows us to determine whether the variations are greater or less than the threshold value, which allows us to determine whether there is facial movement or not. However, within the observations that we have, the average variations between the baseline state and a paralyzed day are sufficient for the development of the algorithm.

8. The term 'automatic' used from line 450 is misleading, because the authors conducted the image analyses post hoc. The term 'unbiased' would be more appropriate.

We added the suggested change.

9. The significance of the section 'Detection of facial paralysis during oral stimulation with sucrose' is unclear. This experiment seems to be an adaptation of a previous work by Delensek and colleagues (Science 2020: doi:

6 10.1126/science.aaz9468.). The authors should revise this section without overstating novelty or consider removing this figure and the accompanying text.

We made the suggested changes.

10. The authors are strongly recommended to send the manuscript to professional scientific/language proof-reading services to improve the writing style. Additionally, the authors are encouraged to unify the styles of figures (e.g. font/size of letters, thickness of lines).

The style and grammar review was carried out, and the styles of the figures and letters were unified.

Minor Concerns:

1. The mouse strain used in the study is described as 'C57BL/6.' However, phenotypes of C57BL/6 mice can vary significantly depending on their substrain, as noted in the literature (e.g., Mekada and Yoshiki 2021; doi: 10.1538/expanim.20-0158) and The Jackson Laboratory website (https://www.jax.org/news-andinsights/jax-blog/2016/june/there-is-no-such-thing-as-a-b6-mouse). The authors should provide information on the substrain and vendor of the mice.

We used C57BL/6J mice (Jackson lab), which was added in the description of the methods 2. In line 89, the authors stated, 'Twelve male and female C57BL/6 mice were used, aged 2 to 6 months.' The authors should discuss whether the differences in sex and age of the mice could impact the results.

An individual analysis was made between males and females. Differences were observed only in the recovery of reversible facial paralysis, where females recovered faster than males, and the developed algorithm can detect this.

3. Why did the authors apply ~8 kPa to induce facial compression, as described in Figure 1C? Is there a rationale or previous literature supporting this procedure? There is no history of the application of 8 kPa. This is the average force applied by the forceps used to cause the injury, recorded by the sensor used. The objective is to document the force used to obtain the reversible facial paralysis model.

4. In lines 42-44, the authors stated, 'However, to apply these strategies, specialized systems are required, as well as specific preparation of study subjects and movement restriction.' Given that the authors' technique also requires specialized devices such as a head-fixation device and treadmill, this statement is 7 incorrect and should be revised.

Thanks for the suggestion. We changed the idea to make what we wanted to say clearer. We made the contributions of our system clear in the introduction and in the abstract.

5. In line 128, the authors wrote, 'Twenty-three measurements,' but it is unclear what was measured 23 times.

Twenty-three measurements were made, one per day from day 2 to 20, three more during the first 24 hours, and the baseline measurement. See the Methods section, page 4, facial paralysis model evaluation.

6. Typo in Figure 4A center: 'divition.' We added the suggested change.
