# Supplementary material for: Facial Paralysis Algorithm: A Tool to Infer Facial Paralysis in Awake Mice
Source: eNeuro. 2025 Feb 28;12(3):ENEURO.0384-24.2025. doi: 10.1523/ENEURO.0384-24.2025 (PMC11963837; doi:10.1523/ENEURO.0384-24.2025)
Supplement: Table 1-1 — Statistical details in the forces applied in the facial nerve. Difference between the forces applied in facial nerve in the crush group (mouse 1 vs. mouse 2 vs mouse 3; Figure 1C). Significance level p<=0.05. Download Table 1-1, RTF file. [file eneuro-12-ENEURO.0384-24.2025-s011.rtf]

Table 1-1

one way ANOVA	
df	F value	p value	
2	0.1081	0.8975	

Statistical details in the forces applied in facial nerve. Difference between the forces applied in facial nerve in the crush group (mouse 1 vs mouse 2 vs mouse 3). Significance level p<=0.05.
