# Supplementary material for: Facial Paralysis Algorithm: A Tool to Infer Facial Paralysis in Awake Mice
Source: eNeuro. 2025 Feb 28;12(3):ENEURO.0384-24.2025. doi: 10.1523/ENEURO.0384-24.2025 (PMC11963837; doi:10.1523/ENEURO.0384-24.2025)
Supplement: Extended Data 1 — Extended data includes a copy of the GitHub repository. It contains a copy of code that charges videos (baseline and transection), processes videos, extracts HOG vectors from videos, calculates a threshold to detect facial paralysis, and applies the FaPA algorithm to both videos. Download Extended Data 1, PDF file. [file eneuro-12-ENEURO.0384-24.2025-s027.pdf]

Extended Data 1

Extended data includes a copy of the GitHub repository. Contains a copy of code that consists in charge videos (baseline and transection), the processing of videos, extract HOGs vectors from videos, calculate threshold to detect facial paralysis and applied FaPA algorithm on both videos.

```
%% Load videos to path
clc, clear all, close all %% Removes system variables and allows the program to start

cd 'C:\Users\warri\Dropbox\yo\paper para trabajar\artículo para trabajr\respuestas\Videos' %% Identify the address where the videos to be used are located
video_baseline=VideoReader('Baseline1.mp4');% reading the video to be used of baseline
video_transection=VideoReader('Paralysis1.mp4');% reading the video to be used of transection day 1
N= video_baseline.NumberOfFrames;% number of frames in baseline video
n= video_transection.NumberOfFrames;% number of frames in transection video
%% Cropping video
img=read(video_baseline,1); % Obtain the first frame from the baseline video
img2=read(video_transection,500);% Obtain the first frame from the transection video
[J,  rect]= imcrop(img);% Cropping the img
coord=rect;% coord save the coordinates of the cropping
% save('coords.mat','coord')% Optional, save coord in archive .mat
close all % Close the image open window
%% Aligned images from transection video to baseline video (Step 1)
figure
imshowpair(img,img2)% Merge of images from baseline and transection video
```

```
h = cpselect(img2,img);% Open window to obtain facial points from images; push ctrl+e and save cpstruct

%% Aligned images from transection video to baseline video (Step 2)
fixedPoints=cpstruct.basePoints;% Obtain point from baseline video
movingPoints=cpstruct.inputPoints;% Obtain point from transection video
tform = fitgeotrans(movingPoints,fixedPoints,'similarity')% Calculate the moves to align images
Jregistered = imwarp(img2,tform,'OutputView',imref2d(size(img2)));% Obtain image from transection video aligned to baseline video
figure
imshowpair(Jregistered,img)% Merge of baseline image and transection aligned image

%% Extract coordinates to anterior and posterior face
close all
img=imcrop(img,coord);% Cropping the image of baseline video
[J,  rect]= imcrop(img);% Cropping the img of anterior area of face
coord2=rect;% coord save the coordinates of the cropping
[J,  rect]= imcrop(img);% Cropping the img of middle area of face
coord3=rect;% coord save the coordinates of the cropping
% save('coordsantandpostface.mat','coord2','coord3')% Optional, save coord2 and coord3 in archive .mat
close all % Close the image open window

%% Extract HOGs from each frame of videos (Baseline)
HOGsba=[]; %Create a new variable to save HOGs of anterior area of face
HOGsbm=[];%Create a new variable to save HOGs of middle area of face
for loop=1:N % loop for extract HOGs from video
```

```
  image=read(video_baseline,loop);% Extract frame to frame from the video

  image=imcrop(image,coord);%image cropping of the face area

  imageanterior=imcrop(image, coord2);% image cropping of the anterior area of the face

  imagemiddle=imcrop(image, coord3);%% image cropping of the middle area of the face

  imageanteriorgray= rgb2gray(imageanterior);%% transform image from RGB to grayscale

  imagemiddlegray= rgb2gray(imagemiddle);%% transform image from RGB to grayscale

  [featureVector,hogVisualization] = extractHOGFeatures(imageanteriorgray, 'CellSize', [32 32],'BlockSize',[1 1] ,'NumBins',8); %% Extract HOGs features from cropping image of anterior area

  [featureVector2,hogVisualization2] = extractHOGFeatures(imagemiddlegray, 'CellSize', [32 32],'BlockSize',[1 1] ,'NumBins',8);%% Extract HOGs features from cropping image of middle area


   HOGsba=[HOGsba;featureVector];% Save HOGs features in variable HOGsba

   HOGsbm=[HOGsbm;featureVector2];%Save HOGs features in variable HOGsbm


end


%% Extract HOGs from each frame of aligned videos (Transection)

HOGsta=[]; %Create a new variable

HOGstm=[];%Create a new variable

for loop=1:n % loop for extract HOGs for video

  image=read(video_transection,loop);% Extract frame to frame from the video

  image = imwarp(image,tform,'OutputView',imref2d(size(img2))); % Aligned image to baseline image

  image=imcrop(image,coord);%image cropping of the face area
```

```
  imageanterior=imcrop(image, coord2);%% image cropping of the anterior area of the face

  imagemiddle=imcrop(image, coord3);%% image cropping of the middle area of the face

  imageanteriorgray= rgb2gray(imageanterior);%% transform image from RGB to grayscale

  imagemiddlegray= rgb2gray(imagemiddle);%% transform image from RGB to grayscale

  [featureVector,hogVisualization] = extractHOGFeatures(imageanteriorgray, 'CellSize', [32 32],'BlockSize',[1 1] ,'NumBins',8); %% Extract HOGs features from cropping image of anterior area

  [featureVector2,hogVisualization2] = extractHOGFeatures(imagemiddlegray, 'CellSize', [32 32],'BlockSize',[1 1] ,'NumBins',8);%% Extract HOGs features from cropping image of middle area


   HOGsta=[HOGsta;featureVector];% Save HOGs features in variable HOGsta

   HOGstm=[HOGstm;featureVector2];%Save HOGs features in variable HOGstm


end


%% Calculate threshold

for loop1=1:length(HOGsba)% loop to obtain differences between frames

   difference1(loop1)=abs(mean(HOGsba(1,:)-HOGsba(loop1,:))); %Create a variable with differences between frame 1 and all the frames in anterior area of baseline video

   difference2(loop1)=abs(mean(HOGsbm(1,:)-HOGsbm(loop1,:))); %Create a variable with differences between frame 1 and all the frames in middle area of baseline video

end

for loop2=1:length(HOGsta)% loop to obtain differences between frames
```

```
    difference3(loop2)=abs(mean(HOGsta(1,:)-HOGsta(loop2,:))); %Create a variable with differences between frame 1 and all the frames in anterior area of transection video

    difference4(loop2)=abs(mean(HOGstm(1,:)-HOGstm(loop2,:))); %Create a variable with differences between frame 1 and all the frames in middle area of transection video

end

max1=max(difference1);% max from baseline anterior area data

min1=min(difference1);% min from baseline anterior area data

max2=max(difference2);% max from baseline middle area data

min2=min(difference2);% min from baseline middle area data

difference1=(difference1-min1)/(max1-min1);% normalized data

difference2=(difference2-min2)/(max2-min2);% normalized data

difference3=(difference3-min1)/(max1-min1);% normalized data

difference4=(difference4-min2)/(max2-min2);% normalized data

theresholdanterior=(mean(difference1)+mean(difference3))/2; % Create a threshold of anterior area

thresholdmiddle=(mean(difference2)+mean(difference4))/2; % Create a threshold of middle area


%% Use FaPDA: Facial paralysis detection algorithm applied in baseline mice


for loop3=1:length(difference1)% loop to obtain differences between frames

if difference1(loop3)<thresholdanterior %conditional to detect frames with values under the threshold

    identityanterior(loop3)=1;% frame without movement

else

    identityanterior(loop3)=0;% frame with movement

end
```

```
if difference2(loop3)<thresholdmiddle %conditional to detect frames with values under the threshold

   identitymiddle(loop3)=1; % frame without movement

else

   identitymiddle(loop3)=0; % frame with movement

end

end


if (double(length(find(identityanterior==1))>(95*n)/100) + double(length(find(identitymiddle==1))>(95*n)/100)) == 2%Detection conditional

   detection=1 %If 95% of the frames are without movement, the system ends up in paralysis.

else

   detection=0 %If 95% of the frames are with movement, the system ends up in without paralysis

end

%% Use FaPDA: Facial paralysis detection algorithm applied in paralyzed mice


for loop4=1:length(difference3)% loop to obtain differences between frames

if difference3(loop4)<thresholdanterior %conditional to detect frames with values under the threshold

   identityanteriort(loop4)=1;% frame without movement

else

   identityanteriort(loop4)=0;% frame with movement

end

if difference4(loop4)<thresholdmiddle %conditional to detect frames with values under the threshold

   identitymiddlet(loop4)=1; % frame without movement

else
```

```
    identitymiddlet(loop4)=0; % frame with movement
end
end

if (double(length(find(identityanteriort==1))>(95*n)/100) + double(length(find(identitymiddlet==1))>(95*n)/100)) == 2%Detection conditional
    detection2=1 %If 95% of the frames are without movement, the algorithm conclude in paralysis
else
    detection2=0 %If 95% of the frames are without movement, the algorithm conclude in without paralysis
end
```
