# Supplementary material for: Facial Paralysis Algorithm: A Tool to Infer Facial Paralysis in Awake Mice
Source: eNeuro. 2025 Feb 28;12(3):ENEURO.0384-24.2025. doi: 10.1523/ENEURO.0384-24.2025 (PMC11963837; doi:10.1523/ENEURO.0384-24.2025)
Supplement: Table 2-1 — Statistical details in whisker movement. Difference between the amplitudes before and after the change point in transection, crush, and sham groups (Figure 2-1C). Significance level p<=0.05. Download Table 2-1, RTF file. [file eneuro-12-ENEURO.0384-24.2025-s012.rtf]

Table 2-1

T-test	
Facial palsy model	df	sd value	p value	
Transection	24	9.128	0.0037	
Crush	14	18.309	0.00067	
Sham	28	13.83	0.00000031	

Statistical details in whisker movement. Difference between the amplitudes before and after the change point in transection, crush and sham groups. Significance level p<=0.05.
