# Supplementary material for: Facial Paralysis Algorithm: A Tool to Infer Facial Paralysis in Awake Mice
Source: eNeuro. 2025 Feb 28;12(3):ENEURO.0384-24.2025. doi: 10.1523/ENEURO.0384-24.2025 (PMC11963837; doi:10.1523/ENEURO.0384-24.2025)
Supplement: Table 3-1 — Statistical details in whisker movement with facial paralysis. Difference in area under the curve between baseline vs. days post facial paralysis in transection, crush, and sham groups (Figure 3A, Figure 3D and Figure 3G). Significance level p<=0.05. Download Table 3-1, RTF file. [file eneuro-12-ENEURO.0384-24.2025-s013.rtf]

Table 3-1

Transection		Crush		Sham	
Analysis: one way ANOVA	
df	F value	p value			df	F value	p value		df	F value	p value	
22	561.408	1E-12			22	10.9544	1.41E-36		22	10.1836569	6.73E-34	
Post hoc Tukey	


Comparation	low confidence
interval	high confidence
interval	

p value		low confidence
interval	high confidence
interval	

p value		low confidence
interval	high confidence
interval	

p value	
.5 hrs	0.7138417	0.78100716	1E-27		0.10539581	1.01729824	0.00204952		-0.1014197	0.09627043	0.99999871	
6 hrs	0.70963214	0.7767976	1E-27		0.11144431	1.1621428	0.00257567		-0.0419804	0.15570974	0.91080611	
Day 1	0.71776382	0.78492928	1E-27		0.11155378	1.16225227	0.00256718		-0.1939849	0.00370524	0.07719326	
Day 2	0.71317778	0.78034324	1E-27		0.09462982	1.1453283	0.00423982		-0.0615361	0.13615399	0.9995366	
Day 3	0.71149686	0.77866232	1E-27		0.10539581	1.1560943	0.00308746		-0.11953	0.07816014	0.9999987	
Day 4	0.71405474	0.7812202	1E-27		0.11896914	1.16966763	0.00204952		-0.0573448	0.14034532	0.99776969	
Day 5	0.70969296	0.77685841	1E-27		0.13520273	1.18590121	0.00123783		-0.009691	0.18799911	0.14591316	
Day 6	0.71384061	0.78100607	1E-27		0.14004315	1.19074164	0.0010619		-0.1348413	0.06284883	0.9997347	
Day 7	0.7133756	0.78054105	1E-27		0.10468558	1.15538407	0.00315342		-0.0719621	0.12572799	0.99999697	
Day 8	0.71431865	0.78148411	1E-27		0.11041299	1.16111147	0.00265692		0.02053692	0.21822701	0.00275958	
Day 9	0.71497784	0.7821433	1E-27		0.079028	1.12972649	0.00663004		-0.1715719	0.02611825	0.52688087	
Day 10	0.71354805	0.78071351	1E-27		0.09086726	1.14156575	0.00472899		-0.1971231	0.00056703	0.05353686	
Day 11	0.71086172	0.77802718	1E-27		0.04650857	1.09720706	0.01602326		-0.1556569	0.04203319	0.91155529	
Day 12	0.70782801	0.77499347	1E-27		-0.104445	0.94625349	0.34646532		-0.1414506	0.0562395	0.99678469	
Day 13	0.71264314	0.7798086	1E-27		-0.0737284	0.97697007	0.21636959		-0.1104895	0.08720056	0.99999871	
Day 14	0.71089786	0.77806332	1E-27		-0.4665345	0.58416403	0.99999871		-0.1935167	0.00417336	0.08137375	
Day 15	0.71188235	0.77904781	1E-27		-0.2483285	0.80237	0.96215568		-0.1140049	0.08368516	0.99999871	
Day 16	0.71241675	0.7795822	1E-27		-0.7951749	0.25552358	0.97154189		-0.1339803	0.06370977	0.9998193	
Day 17	0.71431894	0.7814844	1E-27		-0.7072401	0.34345839	0.99988163		-0.2049204	-0.0072303	0.01973809	
Day 18	0.71193691	0.77910237	1E-27		-0.7388313	0.31186723	0.99860211		-0.1247464	0.07294374	0.99999784	
Day 19	0.71266442	0.77982988	1E-27		-0.5595911	0.49110738	0.99999871		-0.0367969	0.16089321	0.81663512	
Day 20	0.71335228	0.78051774	1E-27		-0.6508563	0.39984219	0.99999856		-0.1424711	0.05521902	0.99556646	

Statistical details in whisker movement with facial paralysis. Difference in area under the curve between baseline vs days post facial paralysis in transection, crush and sham groups. Significance level p<=0.05.
