# Supplementary material for: Facial Paralysis Algorithm: A Tool to Infer Facial Paralysis in Awake Mice
Source: eNeuro. 2025 Feb 28;12(3):ENEURO.0384-24.2025. doi: 10.1523/ENEURO.0384-24.2025 (PMC11963837; doi:10.1523/ENEURO.0384-24.2025)
Supplement: Table 3-2 — Statistical details in the proportion of high and low amplitudes in transection group. Difference between the baseline day vs. days post facial paralysis (Figure 3B). Significance level p<=0.05. Download Table 3-2, RTF file. [file eneuro-12-ENEURO.0384-24.2025-s014.rtf]

Table 3-2

Transection	
Chi-square test	
Comparation	High amplitudes				Low amplitudes			
	p value	percentage	N		p value	percentage	N	
.5 hrs	6.33E-15	0.0443066	1		4.11E-14	99.9556934	2256	
6 hrs	6.66E-15	0.10638298	1		4.36E-14	99.893617	939	
Day 1	9.88E-15	0.4950495	1		6.28E-14	99.5049505	201	
Day 2	9.88E-15	0.4950495	1		6.28E-14	99.5049505	201	
Day 3	9.88E-15	0.4950495	1		6.28E-14	99.5049505	201	
Day 4	9.88E-15	0.4950495	1		6.28E-14	99.5049505	201	
Day 5	9.88E-15	0.4950495	1		6.28E-14	99.5049505	201	
Day 6	9.88E-15	0.4950495	1		6.28E-14	99.5049505	201	
Day 7	9.88E-15	0.4950495	1		6.28E-14	99.5049505	201	
Day 8	9.88E-15	0.4950495	1		6.28E-14	99.5049505	201	
Day 9	9.88E-15	0.4950495	1		6.28E-14	99.5049505	201	
Day 10	9.88E-15	0.4950495	1		6.28E-14	99.5049505	201	
Day 11	9.88E-15	0.4950495	1		6.28E-14	99.5049505	201	
Day 12	9.88E-15	0.4950495	1		6.28E-14	99.5049505	201	
Day 13	9.88E-15	0.4950495	1		6.28E-14	99.5049505	201	
Day 14	9.88E-15	0.4950495	1		6.28E-14	99.5049505	201	
Day 15	9.88E-15	0.4950495	1		6.28E-14	99.5049505	201	
Day 16	9.88E-15	0.4950495	1		6.28E-14	99.5049505	201	
Day 17	9.10E-15	0.4048583	1		5.77E-14	99.5951417	246	
Day 18	9.10E-15	0.4048583	1		5.77E-14	99.5951417	246	
Day 19	9.10E-15	0.4048583	1		5.77E-14	99.5951417	246	
Day 20	9.10E-15	0.4048583	1		5.77E-14	99.5951417	246	

Statistical details in the proportion of high and low amplitudes in transection group.
Difference between the baseline day vs. days post facial paralysis. Significance level p<=0.05.
