# Supplementary material for: Facial Paralysis Algorithm: A Tool to Infer Facial Paralysis in Awake Mice
Source: eNeuro. 2025 Feb 28;12(3):ENEURO.0384-24.2025. doi: 10.1523/ENEURO.0384-24.2025 (PMC11963837; doi:10.1523/ENEURO.0384-24.2025)
Supplement: Table 3-3 — Statistical details in the proportion of high and low amplitudes in the crush group. Difference between the baseline day vs. days post facial paralysis (Figure 3E). Significance level p<=0.05. Download Table 3-3, RTF file. [file eneuro-12-ENEURO.0384-24.2025-s015.rtf]

Table 3-3

Crush	
Chi-square test	
Comparation	High amplitudes				Low amplitudes			
	p value	percentage	N		p value	percentage	N	
.5 hrs	2.32E-09	12.1289228	143		9.37E-09	87.8710772	1036	
6 hrs	0.00014479	30.0469484	128		0.00016274	69.9530516	298	
Day 1	3.10E-13	2.02020202	2		2.54E-12	97.979798	97	
Day 2	3.10E-13	2.02020202	2		2.54E-12	97.979798	97	
Day 3	1.14E-13	1.01010101	1		1.01E-12	98.989899	98	
Day 4	1.14E-13	1.01010101	1		1.01E-12	98.989899	98	
Day 5	1.13E-13	1	1		9.96E-13	99	99	
Day 6	1.13E-13	1	1		9.96E-13	99	99	
Day 7	1.13E-13	1	1		9.96E-13	99	99	
Day 8	1.14E-13	1.01010101	1		1.01E-12	98.989899	98	
Day 9	1.52E-12	3.66972477	4		1.11E-11	96.3302752	105	
Day 10	1.04E-13	0.91743119	1		9.22E-13	99.0825688	108	
Day 11	1.52E-12	3.66972477	4		1.11E-11	96.3302752	105	
Day 12	8.15E-09	13.7614679	15		2.94E-08	86.2385321	94	
Day 13	9.56E-11	8.25688073	9		5.08E-10	91.7431193	100	
Day 14	3.75E-06	22.9357798	25		7.06E-06	77.0642202	84	
Day 15	1.15E-07	17.4311927	19		3.19E-07	82.5688073	90	
Day 16	4.42E-05	27.5229358	30		5.98E-05	72.4770642	79	
Day 17	2.79E-05	26.6055046	29		4.03E-05	73.3944954	80	
Day 18	2.13E-07	18.3486239	20		5.57E-07	81.6513761	89	
Day 19	4.42E-05	27.5229358	30		5.98E-05	72.4770642	79	
Day 20	0.00010599	29.3577982	32		0.00012533	70.6422018	77	

Statistical details in the proportion of high and low amplitudes in crush group. Difference between the baseline day vs. days post facial paralysis. Significance level p<=0.05.
