# Supplementary material for: Facial Paralysis Algorithm: A Tool to Infer Facial Paralysis in Awake Mice
Source: eNeuro. 2025 Feb 28;12(3):ENEURO.0384-24.2025. doi: 10.1523/ENEURO.0384-24.2025 (PMC11963837; doi:10.1523/ENEURO.0384-24.2025)
Supplement: Table 3-4 — Statistical details in the proportion of high and low amplitudes in the sham group. Difference between the baseline day vs. days post facial paralysis (Figure 3H). Significance level p<=0.05. Download Table 3-4, RTF file. [file eneuro-12-ENEURO.0384-24.2025-s016.rtf]

Table 3-4

Sham	
Chi- square test	
Comparation	High amplitudes				Low amplitudes			
	p value	percentage	N		p value	percentage	N	
.5 hrs	0.53933387	48.0716253	698		0.58637691	51.9283747	754	
6 hrs	0.02290352	59.0909091	78		0.38506648	62.5	330	
Day 1	0.32712962	37.5	198		0.04373648	40.9090909	54	
Day 2	0.72501583	41.6666667	55		0.75518889	58.3333333	77	
Day 3	0.99271006	43.9393939	58		0.99353814	56.0606061	74	
Day 4	0.08502074	32.5757576	43		0.12685339	67.4242424	89	
Day 5	0.02182996	28.7878788	38		0.04207221	71.2121212	94	
Day 6	0.65437069	46.969697	62		0.69148364	53.030303	70	
Day 7	0.1352144	34.0909091	45		0.18545059	65.9090909	87	
Day 8	3.78E-05	16.6666667	22		0.00025963	83.3333333	110	
Day 9	0.05330847	56.8181818	75		0.08673064	43.1818182	57	
Day 10	0.00210854	64.3939394	85		0.00642519	35.6060606	47	
Day 11	0.17339698	53.030303	70		0.22753807	46.969697	62	
Day 12	0.73876457	46.2121212	61		0.76753013	53.7878788	71	
Day 13	0.57417904	47.7272727	63		0.61842923	52.2727273	69	
Day 14	0.03070837	58.3333333	77		0.05544505	41.6666667	55	
Day 15	0.35610756	37.8787879	50		0.41336782	62.1212121	82	
Day 16	0.30832505	50.7575758	67		0.36651579	49.2424242	65	
Day 17	0.00095684	65.9090909	87		0.00341451	34.0909091	45	
Day 18	0.49896678	48.4848485	64		0.54896412	51.5151515	68	
Day 19	0.06628696	31.8181818	42		0.10355374	68.1818182	90	
Day 20	0.21233863	52.2727273	69		0.26894746	47.7272727	63	

Statistical details in the proportion of high and low amplitudes in sham group. Difference between the baseline day vs. days post facial paralysis. Significance level p<=0.05.
