# Supplementary material for: Facial Paralysis Algorithm: A Tool to Infer Facial Paralysis in Awake Mice
Source: eNeuro. 2025 Feb 28;12(3):ENEURO.0384-24.2025. doi: 10.1523/ENEURO.0384-24.2025 (PMC11963837; doi:10.1523/ENEURO.0384-24.2025)
Supplement: Table 4-1 — Statistical details in the differences between frames in the transection group. Difference between the first frame with the others in the video, comparison between baseline vs days post facial paralysis (Figure 4B). Significance level p<=0.05. Download Table 4-1, RTF file. [file eneuro-12-ENEURO.0384-24.2025-s017.rtf]

Table 4-1

Transection posterior area		Transection middle area		Transection anterior area	
Analysis: one way ANOVA	
df	F value	p value			df	F value	p value		df	F value	p value	
22	1.98568404	0.02501113			22	3.01086235	0.00080586		22	5.16887522	1.37E-06	
Post hoc Tukey	


Comparation	low confidence
interval	high confidence
interval	

p value		low confidence
interval	high confidence
interval	

p value		low confidence
interval	high confidence
interval	

p value	
.5 hrs	-0.018964	1.3953445	0.06506211		0.27230543	1.4851494	0.00026894		0.39573887	1.3237196	1.29E-06	
6 hrs	-0.0519695	1.3623389	0.10079956		0.32170647	1.5345504	9.48E-05		0.1706228	1.0986035	0.00074793	
Day 1	-0.0756797	1.3386288	0.13562822		0.28836226	1.5012062	0.00019199		0.19813761	1.1261183	0.00035543	
Day 2	0.06866986	1.4829783	0.01811275		0.31953555	1.5323794	9.92E-05		0.45431277	1.3822935	1.55E-07	
Day 3	0.02064103	1.4349494	0.03720494		0.29330754	1.5061514	0.00017298		0.46798274	1.3959634	7.01E-08	
Day 4	-0.0625867	1.3517218	0.1153489		0.13537121	1.3482151	0.00431938		0.47751591	1.4054966	2.71E-08	
Day 5	-0.152395	1.2619134	0.31449121		0.2667529	1.4795969	0.00030207		0.33906248	1.2670432	6.89E-06	
Day 6	-0.2152659	1.1990426	0.53312689		0.1583572	1.371201	0.00275307		0.45332471	1.3813055	1.63E-07	
Day 7	0.06507176	1.4793801	0.01914364		0.13151646	1.3443604	0.00465455		0.38073978	1.3087205	2.04E-06	
Day 8	0.10954159	1.52385	0.0095207		0.27245194	1.4852958	0.00026815		0.36002603	1.2880068	3.76E-06	
Day 9	0.03835154	1.45266	0.02867355		0.34089684	1.5537407	6.30E-05		0.35821596	1.2861967	3.96E-06	
Day 10	0.0938822	1.5081906	0.01221775		0.18920183	1.4020457	0.00148738		0.43966916	1.3676499	2.94E-07	
Day 11	0.00172216	1.4160306	0.04879763		0.13790977	1.3507537	0.00411144		0.43063542	1.3586161	4.10E-07	
Day 12	-0.118279	1.2960293	0.22166331		0.24242646	1.4552703	0.00050109		0.274905	1.2028857	4.25E-05	
Day 13	-0.3050938	1.1092145	0.84492576		0.15158343	1.3644273	0.00314647		0.29383722	1.221818	2.49E-05	
Day 14	0.20779371	1.6221021	0.00185547		0.04883152	1.2616754	0.02167619		0.50240517	1.4303858	-4.32E-08	
Day 15	0.01864469	1.4329531	0.03829856		0.01077449	1.2236184	0.04181074		0.33957621	1.2675569	6.79E-06	
Day 16	-0.1291753	1.2851331	0.2489731		0.19001663	1.4028605	0.00146319		0.39186338	1.3198441	1.46E-06	
Day 17	0.10062999	1.5149384	0.01097753		0.11361337	1.3264573	0.00656569		0.40859351	1.3365742	8.66E-07	
Day 18	-0.0468044	1.3675041	0.09429703		0.21125758	1.4241015	0.00095097		0.30926922	1.23725	1.61E-05	
Day 19	0.00905287	1.4233613	0.04396841		-0.015891	1.1969529	0.0646777		0.34196219	1.2699429	6.33E-06	
Day 20	0.03635639	1.4506648	0.02953629		0.10167348	1.3145174	0.00823355		0.4378148	1.3657955	3.15E-07	

Statistical details in the differences between frames in transection group. Difference between the first frame with the others in the video, comparation between baseline vs days post facial paralysis. Significance level p<=0.05.
