# Supplementary material for: Facial Paralysis Algorithm: A Tool to Infer Facial Paralysis in Awake Mice
Source: eNeuro. 2025 Feb 28;12(3):ENEURO.0384-24.2025. doi: 10.1523/ENEURO.0384-24.2025 (PMC11963837; doi:10.1523/ENEURO.0384-24.2025)
Supplement: Table 4-2 — Statistical details in the differences between frames in the crush group. Difference between the first frame with the others in the video, comparison between baseline vs days post facial paralysis (Figure 4B). Significance level p<=0.05. Download Table 4-2, RTF file. [file eneuro-12-ENEURO.0384-24.2025-s018.rtf]

Table 4-2

Crush posterior area		Crush middle area		Crush anterior area	
Analysis: one way ANOVA	
df	F value	p value			df	F value	p value		df	F value	p value	
22	2.12548065	0.01560637			22	3.76091599	7.52E-05		22	4.72901535	4.50E-06	
Post hoc Tukey	


Comparation	low confidence
interval	high confidence
interval	

p value		low confidence
interval	high confidence
interval	

p value		low confidence
interval	high confidence
interval	

p value	
.5 hrs	-0.5589215	1.0581894	0.99985915		0.36278164	1.5345566	3.00E-05		0.08144373	1.3261409	0.01247929	
6 hrs	-0.1639256	1.4531853	0.28785354		0.32556146	1.4973364	6.85E-05		0.20385897	1.4485561	0.00122909	
Day 1	-0.3247579	1.2923529	0.78269285		0.32208389	1.4938588	7.40E-05		0.32631481	1.5710119	0.00010264	
Day 2	-0.0451583	1.5719526	0.08570348		0.26873499	1.44051	0.00023797		0.23976284	1.4844599	0.00060093	
Day 3	-0.2585768	1.3585341	0.57443261		0.3022337	1.4740087	0.00011449		0.24808675	1.4927838	0.00050832	
Day 4	-0.5283838	1.088727	0.99923044		0.28739589	1.4591708	0.0001584		0.35177535	1.5964725	6.05E-05	
Day 5	-0.2451534	1.3719575	0.52988642		0.34810841	1.5198834	4.15E-05		0.22293895	1.4676361	0.00084166	
Day 6	-0.1841391	1.4329717	0.34135166		0.29827052	1.4700456	0.00012487		0.29208148	1.5367786	0.00020783	
Day 7	-0.0282502	1.5888608	0.0703482		0.27919728	1.4509723	0.00018951		0.23399103	1.4786881	0.00067479	
Day 8	-0.3149961	1.3021147	0.75473726		0.32449281	1.4962678	7.01E-05		0.25244755	1.4971447	0.00046547	
Day 9	-0.0698816	1.5472293	0.11323776		0.22321588	1.3949909	0.00063397		0.26362765	1.5083247	0.00037116	
Day 10	-0.6981545	0.91895634	0.99999875		0.1967591	1.3685341	0.00111015		-0.2323326	1.0123645	0.71505177	
Day 11	-0.1100537	1.5070572	0.17316373		0.17030233	1.3420773	0.00192822		-0.2172025	1.0274945	0.65244621	
Day 12	0.08740407	1.704515	0.0160597		0.2402696	1.4120445	0.00044012		-0.0456387	1.1990583	0.1006551	
Day 13	-0.2775111	1.3395998	0.63714176		0.17071962	1.3424946	0.0019117		-0.1807303	1.0639668	0.49590263	
Day 14	-0.3738774	1.2432334	0.89777726		0.13132381	1.3030988	0.00427305		-0.2501896	0.99450749	0.78349721	
Day 15	-0.2611921	1.3559188	0.58313072		0.06326246	1.2350374	0.01608122		-0.1844164	1.0602807	0.51157218	
Day 16	-0.2477484	1.3693624	0.5384711		0.02676553	1.1985404	0.03136139		0.0128715	1.2575686	0.04058885	
Day 17	-0.4555275	1.1615833	0.98577821		-0.0097314	1.1620436	0.05891329		-0.1489977	1.0956994	0.36813438	
Day 18	-0.3889422	1.2281687	0.92333037		-0.0887544	1.0830207	0.19599441		-0.1461186	1.0985785	0.35739094	
Day 19	-0.1789375	1.4381733	0.32706818		-0.0080537	1.1637213	0.05728279		-0.0361619	1.2085352	0.087519	
Day 20	-0.1164183	1.5006926	0.1845856		0.0623169	1.2340919	0.01636869		0.07497919	1.3196763	0.01400852	

Statistical details in the differences between frames in crush group. Difference between the first frame with the others in the video, comparation between baseline vs days post facial paralysis. Significance level p<=0.05.
