# Supplementary material for: Facial Paralysis Algorithm: A Tool to Infer Facial Paralysis in Awake Mice
Source: eNeuro. 2025 Feb 28;12(3):ENEURO.0384-24.2025. doi: 10.1523/ENEURO.0384-24.2025 (PMC11963837; doi:10.1523/ENEURO.0384-24.2025)
Supplement: Table 4-3 — Statistical details in the differences between frames in the sham group. Difference between the first frame with the others in the video, comparison between baseline vs days post facial paralysis (Figure 4-2A). Significance level p<=0.05. Download Table 4-3, RTF file. [file eneuro-12-ENEURO.0384-24.2025-s019.rtf]

Table 4-3

Sham posterior area		Sham middle area		Sham anterior area	
Analysis: one way ANOVA	
df	F value	p value			df	F value	p value		df	F value	p value	
22	0.44879752	0.97764826			22	1.54423678	0.10641832		22	2.19553185	0.0123127	
Post hoc Tukey	


Comparation	low confidence
interval	high confidence
interval	

p value		low confidence
interval	high confidence
interval	

p value		low confidence
interval	high confidence
interval	

p value	
.5 hrs	-1.0951746	0.6982885	0.99999827		-0.7443456	0.81382954	0.99999875		-0.787832	0.78234756	0.99999875	
6 hrs	-0.8135519	0.97991121	0.99999875		-0.9208729	0.63730216	0.99999875		-0.7956059	0.77457368	0.99999875	
Day 1	-0.9362071	0.857256	0.99999875		-0.9883462	0.56982887	0.99998391		-1.0234889	0.5466907	0.9998908	
Day 2	-0.8205403	0.9729228	0.99999875		-0.8738245	0.68435061	0.99999875		-0.9089946	0.66118503	0.99999875	
Day 3	-1.0024329	0.79103017	0.99999875		-0.7636162	0.79455888	0.99999875		-0.9159247	0.65425491	0.99999875	
Day 4	-1.08132	0.712143	0.99999863		-0.9329022	0.62527293	0.99999869		-0.6968494	0.87333024	0.99999875	
Day 5	-0.7557613	1.0377018	0.99999875		-1.0035428	0.55463237	0.99995184		-0.8268335	0.7433461	0.99999875	
Day 6	-0.9121696	0.88129348	0.99999875		-0.9547927	0.60338235	0.99999809		-0.959147	0.61103261	0.99999821	
Day 7	-0.8213451	0.97211802	0.99999875		-1.2257459	0.33242923	0.8357116		-0.6340872	0.93609238	0.99999869	
Day 8	-0.9110919	0.88237125	0.99999875		-0.817365	0.7408101	0.99999875		-0.9686954	0.60148418	0.99999738	
Day 9	-0.9415035	0.85195965	0.99999875		-0.9288681	0.62930697	0.99999869		-0.8084906	0.76168895	0.99999875	
Day 10	-1.085415	0.70804811	0.99999857		-1.1714947	0.38668036	0.94082814		-0.9846116	0.58556801	0.99999297	
Day 11	-0.9212912	0.87217194	0.99999875		-1.1300827	0.42809236	0.98004013		-0.9870934	0.58308619	0.9999916	
Day 12	-0.92792	0.86554313	0.99999875		-1.0338666	0.52430844	0.9996658		-1.1847303	0.38544923	0.93504006	
Day 13	-0.9780067	0.81545639	0.99999875		-1.0628071	0.495368	0.99847245		-0.8726939	0.69748569	0.99999875	
Day 14	-0.7969792	0.99648392	0.99999875		-1.1832244	0.37495071	0.92362344		-0.9160974	0.65408218	0.99999875	
Day 15	-0.9790111	0.81445205	0.99999875		-0.753053	0.80512214	0.99999875		-0.9150201	0.65515947	0.99999875	
Day 16	-0.7794061	1.0140569	0.99999875		-1.115078	0.44309711	0.98760289		-1.2583408	0.31183881	0.77255231	
Day 17	-1.1229515	0.67051154	0.99999368		-1.2866516	0.27152342	0.65115482		-1.2162427	0.35393691	0.87951624	
Day 18	-0.8742496	0.91921347	0.99999875		-1.3377128	0.22046238	0.47643399		-1.3639631	0.20621645	0.42449856	
Day 19	-0.7459262	1.0475368	0.99999875		-0.7697285	0.78844655	0.99999875		-0.6055963	0.96458328	0.99999785	
Day 20	-0.9814837	0.81197941	0.99999875		-1.0629236	0.49525154	0.99846399		-1.4006104	0.16956913	0.31552583	

Statistical details in the differences between frames in sham group. Difference between the first frame with the others in the video, comparation between baseline vs days post facial paralysis. Significance level p<=0.05.
