# Supplementary figures and images for: Facial Paralysis Algorithm: A Tool to Infer Facial Paralysis in Awake Mice
Source: eNeuro. 2025 Feb 28;12(3):ENEURO.0384-24.2025. doi: 10.1523/ENEURO.0384-24.2025 (PMC11963837; doi:10.1523/ENEURO.0384-24.2025)

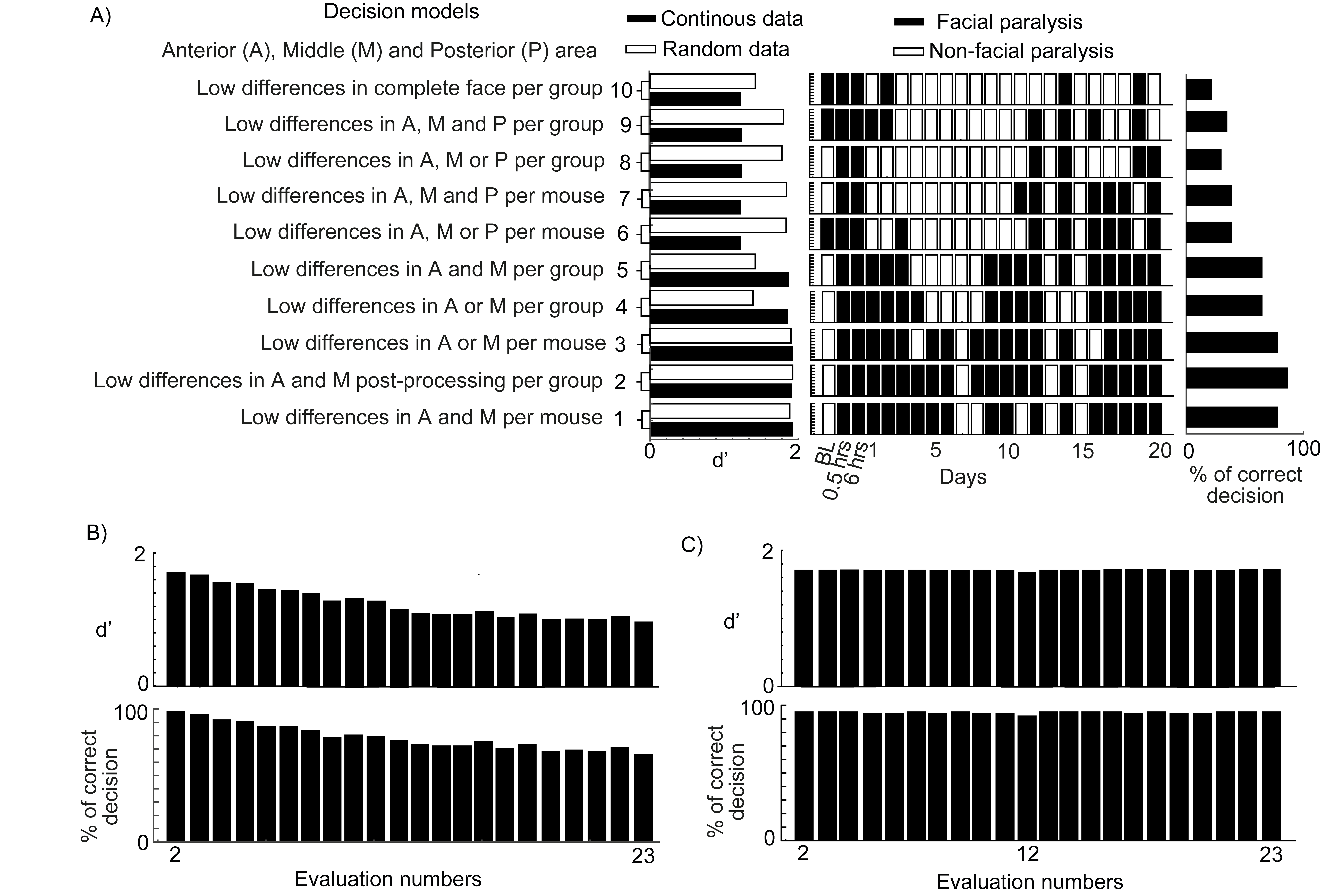

Supplement: Figure 5-2 — Facial paralysis algorithm efficiency. A) The left panel shows the level of efficiency of the different algorithms designed to detect facial paralysis in continuous and random data. The middle panel shows the application of each algorithm to infer facial paralysis in a characteristic mouse. The right panel shows the percentage of correct decisions made for each algorithm. The rectangle indicates the designed algorithm with the highest efficiency in predicting paralysis. B) The upper panel shows the efficiency of the algorithm using different amounts of post-facial injury assessments averaged together; each bar indicates the number of assessments used. The low panel shows the percentage of correct decisions made from each algorithm. C) The upper panel shows the efficiency of the algorithm when averaging the baseline with one post-facial injury assessment; each bar indicates the assessment used with the baseline. The low panel shows the percentage of correct decisions made from each algorithm. Download Figure 5-2, TIF file. [file eneuro-12-ENEURO.0384-24.2025-s007.tif]

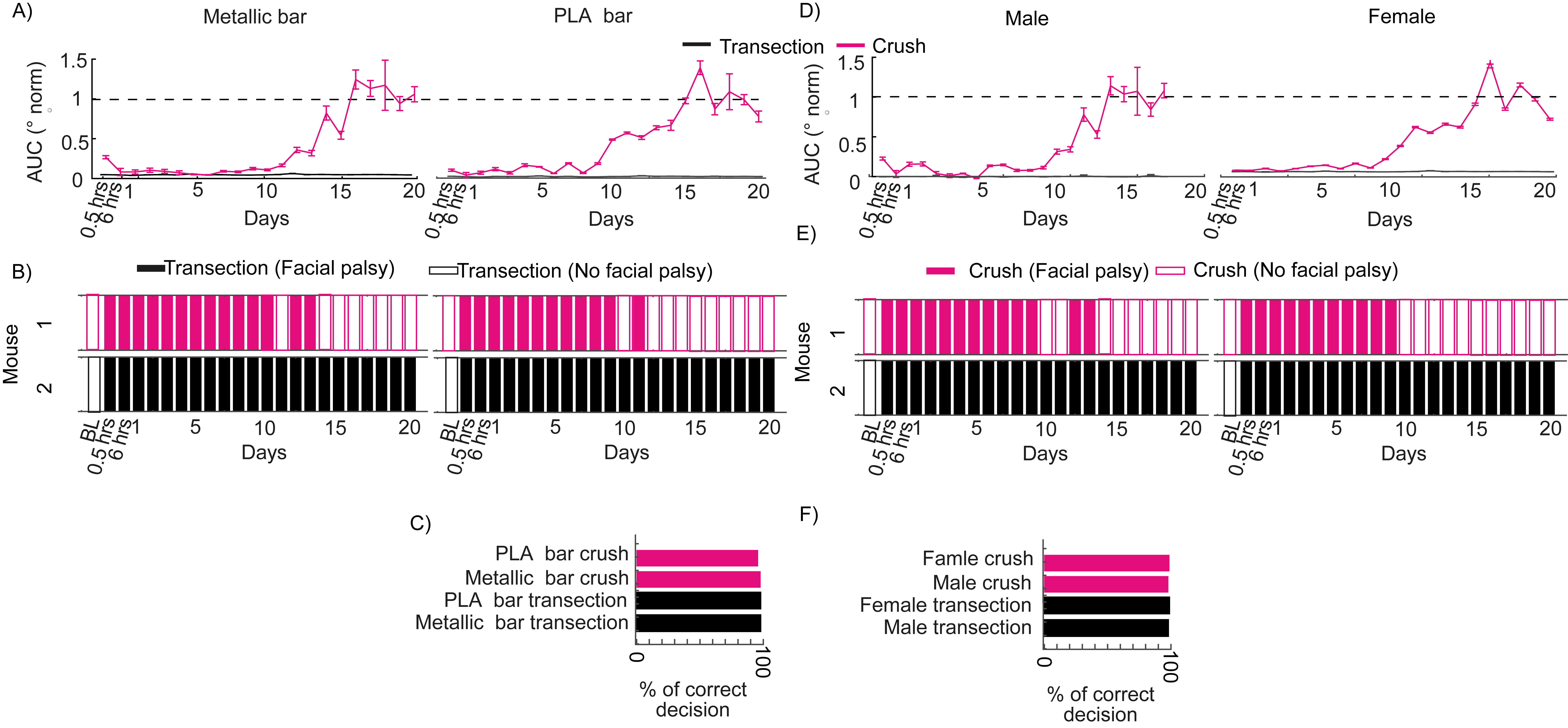

Supplement: Figure 6-1 — Facial paralysis detection between fixed head restraint systems and between sexes. A) Area under the curve of the whisker movement cycle over 20 years of transection and compression paralysis in two different fixed head restraint systems (metal bar and bar made of PLA). The dotted line indicates the baseline value (n = 3 per group). B) Use of the FaPA for the detection of facial paralysis in a mouse characteristic of the compression and transection group of each fixation system. C) Percentage of correct decisions made by the FaPA for the transection and compression group (n = 3 per group). D) Area under the curve of the whisker movement cycle over 20 years of transection and compression paralysis between males and females (n = 3 per group). The dotted line indicates the baseline value. E) Use of the FaPA for the detection of facial paralysis in a mouse characteristic of the compression and transection group in males and females. C) Percentage of correct decisions made by FaPA for the transection and compression group (n = 3 per group). Detailed statistics in Extended Data Table 6-1, Table 6-2, and Table 6-3. Download Figure 6-1, TIF file. [file eneuro-12-ENEURO.0384-24.2025-s008.tif]

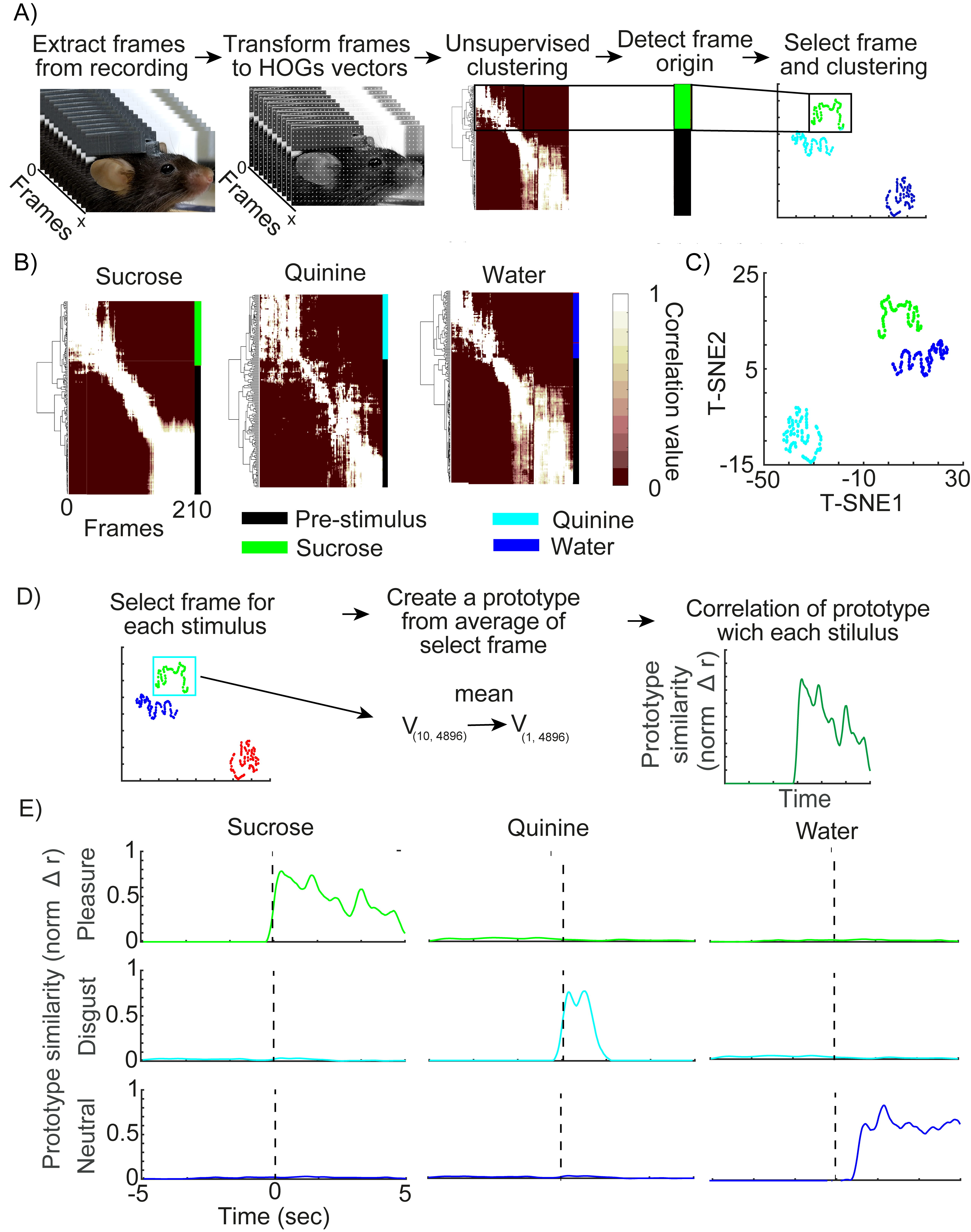

Supplement: Figure 6-2 — Gustatory stimuli induce facial expressions. A) Schematic of the analysis process of the video recordings to detect facial expressions. B) Heat map of the correlation of the frames of 3 stimuli (n = 210 in one mouse) before and after the release of sucrose, water, and quinine, sorted by clusters. C) Grouping by clusters of the frames after oral stimulation with sucrose, water, and quinine. D) Schematic of the creation of pleasant, unpleasant, and neutral prototypes. E) Similarity of the prototypes with the moments of oral stimulation with the different solutions (sucrose, quinine and water) in three mice. The dotted line at 0 shows the moment of the release of solutions. Detailed statistics in Extended Data Table 6-4. Download Figure 6-2, TIF file. [file eneuro-12-ENEURO.0384-24.2025-s009.tif]
