# Supplementary material for: Facial Paralysis Algorithm: A Tool to Infer Facial Paralysis in Awake Mice
Source: eNeuro. 2025 Feb 28;12(3):ENEURO.0384-24.2025. doi: 10.1523/ENEURO.0384-24.2025 (PMC11963837; doi:10.1523/ENEURO.0384-24.2025)
Supplement: Table 6-1 — Statistical details in whisker movement with facial paralysis in two different bars and sex. Difference in whisker movements between bars and sex in transection and crush models (Figure 6-1A and Figure 6-1D). Significance level p<=0.05. Download Table 6-1, RTF file. [file eneuro-12-ENEURO.0384-24.2025-s020.rtf]

Table 6-1

Analysis: one way ANOVA (two bars)	
df	F value	p value		
3	16.0912	2.37E-08		
Post hoc Tukey	
Comparation	Low confidence interval	High confidence interval	p value	
Metallic bar with transection vs PLA bar with transection	
0.0086	
0.2230	
.9996	
Metallic bar with transection vs Metallic bar with crush	
-0.3144	
-0.1	
0.0013	
Metallic bar with transection vs PLA bar with crush	
-0.4556	
-0.2411	
1.788 E-06	
PLA bar with transection vs Metallic bar with crush	
-0.3230	
-0.1085	
9.225 E-04	
PLA bar with transection vs PLA bar with crush	
-0.6786	
-0.2497	
1.1515 E-06	
Metallic bar with crush vs PLA bar with crush	
-0.1411	
0.0733	
0.3172	
Analysis: one way ANOVA (sex)	
df	F value	p value		
3	13.11	4.22E-07		
Post hoc Tukey	
Comparation	Low confidence interval	High confidence interval	p value	
Male transection vs male crush	-0.2917	-0.0725	0.0042	
Male transection vs female transection	
0.0090	
0.2281	
0.9995	
Male transection vs female crush	
-0.4186	
-0.1994	
1.77 E-05	
Male crush vs female transection	
-0.3007	
-0.0815	
0.0030	
Male crush vs female crush	-0.1269	0.0923	0.4316	
Female transection vs female crush	
-0.4276	
-0.2084	
1.15 E-05	

Statistical details in whisker movement with facial paralysis in two different bars and sex. Difference in whisker movements between bars and sex in transection and crush models. Significance level p<=0.05.
