# Supplementary material for: Facial Paralysis Algorithm: A Tool to Infer Facial Paralysis in Awake Mice
Source: eNeuro. 2025 Feb 28;12(3):ENEURO.0384-24.2025. doi: 10.1523/ENEURO.0384-24.2025 (PMC11963837; doi:10.1523/ENEURO.0384-24.2025)
Supplement: Table 6-2 — Statistical details in whisker movement with facial paralysis between systems. Difference in area under the curve between baseline vs days post facial paralysis in transection, and crush groups. MBT = metallic bar in the transection group, PBT = PLA bar in the transection group, MBC = metallic bar in the crush group, and PBC = PLA bar in the crush group (Figure 6-1A). Significance level p<=0.05. Download Table 6-2, RTF file. [file eneuro-12-ENEURO.0384-24.2025-s021.rtf]

Table 6-2

Analysis: two-way ANOVA	Post hoc Tukey p value	
Comparation day	df	F value	p value	MBT vs PBT	MBT vs MBC	MBT vs PBC	PBT vs MBC	PBT vs PBC	MBC vs PBC	
Baseline	3	1.44211	5.4014e-21	0.9999	0.9898	0.0899	0.9876	0.0876	0.7897	
.5 hrs	3	55.5729	9.3497e-29	0.9999	0.9980	0.9995	0.9999	0.9999	0.9920	
6 hrs	3	1.85239	0.13771488	0.3357	0.9996	0.9596	0.2847	0.1302	0.9785	
Day 1	3	1.85193	0.13784610	0.1281	0.9871	0.8982	0.2508	0.4464	0.9836	
Day 2	3	4.11889	0.00696843	0.9999	0.0397	0.9589	0.0356	0.0087	0.9674	
Day 3	3	2.37709	0.07006834	0.9996	0.1271	0.9990	0.1022	0.9955	0.1703	
Day 4	3	1.40376	0.24181458	0.9994	0.2607	0.8634	0.3178	0.9103	0.7220	
Day 5	3	9.89379	3.1047e-06	0.9860	0.0179	1.62e-05	0.0479	8.16e-05	0.2922	
Day 6	3	4.32196	0.00530865	0.9919	0.0064	0.5222	0.0162	0.7027	0.2360	
Day 7	3	11.3876	4.3648e-07	0.9965	1.53e-05	0.0015	4.27e-05	0.0035	0.7098	
Day 8	3	9.31694	6.6557e-06	0.9174	6.99e-06	0.3785	0.0001	0.7748	0.0061	
Day 9	3	14.1099	1.1996e-08	0.9602	3.26e-06	2.09e-05	3.76e-05	0.0001	0.9836	
Day 10	3	45.4988	5.1835e-24	0.9971	3.66e-05	0.0001	9.23e-05	8.54e-24	1.05e-07	
Day 11	3	85.6030	1.6306e-39	0.9999	7.40e-08	0.0001	7.40e-08	0.0001	1.41e-15	
Day 12	3	40.8135	5.2247e-22	0.9998	2.43e-16	4.10e-12	1.31e-16	2.48e-12	0.6485	
Day 13	3	42.8774	2.9132e-23	0.9999	6.41e-11	9.10e-19	4.08e-11	4.76e-19	0.1471	
Day 14	3	29.9438	5.8107e-17	0.9993	1.87e-15	0.0033	6.16e-16	0.0021	1.75e-05	
Day 15	3	53.8498	2.8864e-28	0.9999	2.79e-17	8.98e-21	1.86e-17	5.72e-21	0.8639	
Day 16	3	39.2329	1.1816e-21	0.9999	2.17e-17	6.14e-10	2.17e-17	6.14e-10	0.1442	
Day 17	3	45.6461	1.2470e-24	0.9999	0.0001	8.62e-06	0.0001	9.33e-06	1.17e-06	
Day 18	3	5.09200	0.00186009	0.9999	0.0075	0.0165	0.0070	0.0152	0.6736	
Day 19	3	40.5769	2.7441e-22	0.9999	3.55e-19	1.71e-08	1.97e-19	1.22-08	0.0118	
Day 20	3	40.4189	3.2546e-22	0.9999	2.02e-21	0.0004	1.12e-21	0.0003	1.88e-07	

Statistical details in whisker movement with facial paralysis between systems. Difference in area under the curve between baseline vs days post facial paralysis in transection, and crush groups. MBT=metallic bar in transection group, PBT= PLA bar in transection group, MBC= metallic bar in crush group and PBC= PLA bar in crush group. Significance level p<=0.05.
