# Supplementary material for: Facial Paralysis Algorithm: A Tool to Infer Facial Paralysis in Awake Mice
Source: eNeuro. 2025 Feb 28;12(3):ENEURO.0384-24.2025. doi: 10.1523/ENEURO.0384-24.2025 (PMC11963837; doi:10.1523/ENEURO.0384-24.2025)
Supplement: Table 6-3 — Statistical details in whisker movement with facial paralysis between sex. Difference in area under the curve between baseline vs days post facial paralysis in transection, and crush groups. MT = male in transection group, MC = male in crush group, FT = female in transection group and FC = female in crush group (Figure 6-1D). Significance level p<=0.05. Download Table 6-3, RTF file. [file eneuro-12-ENEURO.0384-24.2025-s022.rtf]

Table 6-3

Analysis: two-way ANOVA	Post hoc Tukey p value	
Comparation day	df	F value	p value	MT vs MC	MT vs FT	MT vs FC	MC vs FT	MC vs FC	FT vs FC	
Baseline	3	37.4420	0.40142600	0.2823	0.9995	0.2031	0.2001	0.0970	0.6620	
.5 hrs	3	55.8648	0.55899054	0.9999	0.9801	0.9995	0.9999	0.9999	0.9963	
6 hrs	3	2.04969	0.10698452	0.3325	0.9999	0.9592	0.1625	0.1280	0.9995	
Day 1	3	2.11733	0.09810744	0.1280	0.9996	0.8982	0.1280	0.4463	0.8982	
Day 2	3	3.99687	0.08820473	0.4407	0.9930	0.9593	0.0530	0.0901	0.9328	
Day 3	3	2.53986	0.05665895	0.1277	0.9937	0.9990	0.0672	0.9955	0.9771	
Day 4	3	1.31478	0.26970251	0.2575	0.9975	0.8627	0.3948	0.7197	0.9539	
Day 5	3	11.2561	5.1836e-07	0.0175	0.5011	0.1545	0.5496	0.2903	0.9939	
Day 6	3	3.60912	0.01377226	0.0058	0.9993	0.5151	0.2390	0.2294	0.9999	
Day 7	3	12.2094	1.4957e-07	1.69e-05	0.9519	0.1661	9.18e-06	0.7126	0.1042	
Day 8	3	9.61325	4.4971e-06	6.06e-06	0.9896	0.3733	7.71e-05	0.0057	0.0061	
Day 9	3	14.9203	4.2259e-09	2.54e-06	0.9999	1.61e-05	1.22e-05	0.9832	6.99e-05	
Day 10	3	46.6848	1.5844e-24	3.46e-05	0.9939	0.0001	3.51e-05	9.70e-08	0.0001	
Day 11	3	886.938	5.7298e-40	7.97e-08	0.9984	0.0001	1.69e-08	1.65e-15	0.0001	
Day 12	3	41.1923	3.5195e-22	2.75e-16	0.9991	4.47e-12	6.01e-17	0.6496	1.29e-12	
Day 13	3	41.6773	1.0409e-22	6.94e-11	0.9999	1.05e-18	1.85e-10	0.1482	4.36e-18	
Day 14	3	29.6847	7.8136e-17	1.62e-15	0.9999	0.0032	1.26e-15	1.67e-05	0.0029	
Day 15	3	53.6997	3.3514e-28	2.61e-17	0.9999	8.34e-21	2.55e-17	0.8636	8.15e-21	
Day 16	3	39.1616	1.2773e-21	2.04e-17	0.9999	5.95e-10	2.61e-17	0.1437	7.05e-10	
Day 17	3	45.6733	1.2121e-24	0.0001	0.9999	8.65e-06	0.0001	1.17e-06	8.98e-06	
Day 18	3	5.06214	0.00193659	0.0074	0.9999	0.0016	0.0074	0.6734	0.0016	
Day 19	3	40.5574	2.8026e-22	3.47e-19	0.9999	1.69e-08	2.09e-19	0.0118	1.27e-08	
Day 20	3	40.4029	3.3127e-22	1.48e-21	0.9999	0.0004	1.30e-21	1.81e-07	0.0004	

Statistical details in whisker movement with facial paralysis between sex. Difference in area under the curve between baseline vs days post facial paralysis in transection, and crush groups. MT=male in transection group, MC= male in crush group, FT= female in transection group and FC= female in crush group.
Significance level p<=0.05.
