# Supplementary material for: Facial Paralysis Algorithm: A Tool to Infer Facial Paralysis in Awake Mice
Source: eNeuro. 2025 Feb 28;12(3):ENEURO.0384-24.2025. doi: 10.1523/ENEURO.0384-24.2025 (PMC11963837; doi:10.1523/ENEURO.0384-24.2025)
Supplement: Table 6-4 — Statistical details in facial expression before and after oral stimulation with solutions. Comparison between 10 seconds pre- and post-stimulation with sucrose, quinine, or water using the pleasure, disgust, and neutral prototype (Figure 6-2E). Significance level p<=0.05. Download Table 6-4, RTF file. [file eneuro-12-ENEURO.0384-24.2025-s023.rtf]

Table 6-4

T-test	
facial palsy model	df	sd value	p value	
sucrose vs pleasure	899	0.18200533	5.60E-86	
quinine vs disgust	899	0.2324	1.20E-27	
water vs neutral	899	0.2074	5.21E-50	

Statistical details in facial expression before and after oral stimulation with solutions. Comparation between 10 seconds pre and post stimulation with sucrose, quinine or water using the pleasure, disgust and neutral prototype. Significance level p<=0.05.
