# Supplementary material for: Facial Paralysis Algorithm: A Tool to Infer Facial Paralysis in Awake Mice
Source: eNeuro. 2025 Feb 28;12(3):ENEURO.0384-24.2025. doi: 10.1523/ENEURO.0384-24.2025 (PMC11963837; doi:10.1523/ENEURO.0384-24.2025)
Supplement: Table 6-5 — Statistical details in facial expression between baseline vs facial paralysis. The similarity of pleasure prototype for 10 seconds after oral stimulation with sucrose between baseline, day 1, and day 20 post facial paralysis in transection and crush group (Figure 6D and Figure 6E). Significance level p<=0.05. Download Table 6-5, RTF file. [file eneuro-12-ENEURO.0384-24.2025-s024.rtf]

Table 6-5

T-test	
Comparation	sd	df	p value	
Transection day 1	0.208	899	1E-31	
Transection day 20	0.1878	899	1.00E-36	
				
Crush day 1	0.2151	899	3.05E-13	
Crush day 20	0.1721	899	1.00E-36	

Statistical details in facial expression between baseline vs facial paralysis. Similarity of pleasure prototype for 10 second after oral stimulation with sucrose between baseline, day 1 and day 20 post facial paralysis in transection and crush group. Significance level p<=0.05.
