# Supplementary material for: Facial Paralysis Algorithm: A Tool to Infer Facial Paralysis in Awake Mice
Source: eNeuro. 2025 Feb 28;12(3):ENEURO.0384-24.2025. doi: 10.1523/ENEURO.0384-24.2025 (PMC11963837; doi:10.1523/ENEURO.0384-24.2025)
Supplement: Table 7-1 — Statistical details in population neuronal activity in ALM pre- and post-facial expression. Differences between z-score pre- and post-oral stimulation with sucrose in population neuronal activity of two mice (Figure 7B and Figure 7C). Significance level p<=0.05. Download Table 7-1, RTF file. [file eneuro-12-ENEURO.0384-24.2025-s025.rtf]

Table 7-1

Wilcoxon signed rank-test	
	z value	p value	N of mice	N of neurons	
Transection baseline	-3.7642	1.67E-04	2	21, mouse1= 12, mouse2= 9	
Transection day 1	1.5722	0.11591433	2	20, mouse1= 11, mouse2= 9	
Transection day 20	-1.2612	0.20723854	2	17, mouse1= 9, mouse2= 8	
					
Crush baseline	8.4251	3.60E-17	2	24, mouse1= 12, mouse2= 12	
Crush day 1	-0.6008	0.54799886	2	22, mouse1= 12, mouse2= 10	
Crush day 20	-11.6501	2.29E-31	2	15, mouse1= 9, mouse2= 6	

Statistical details in population neuronal activity in ALM pre and post facial expression. Differences between z-score pre and post oral stimulation with sucrose in population neuronal activity of two mice. Significance level p<=0.05.
