# Supplementary material for: Facial Paralysis Algorithm: A Tool to Infer Facial Paralysis in Awake Mice
Source: eNeuro. 2025 Feb 28;12(3):ENEURO.0384-24.2025. doi: 10.1523/ENEURO.0384-24.2025 (PMC11963837; doi:10.1523/ENEURO.0384-24.2025)
Supplement: Table 7-2 — Statistical details in population neuronal activity in ALM pre- and post-control events. Differences between z-score pre- and post-walking and activation of a stepper motor in population neuronal activity of two mice (Figure 7-1A and Figure 7-1B). Significance level p<=0.05. Download Table 7-2, RTF file. [file eneuro-12-ENEURO.0384-24.2025-s026.rtf]

Table 7-2

Wilcoxon signed rank-test	
Mouse walking		Stepper motor			
	z value	p value			z value	p value	N of mice	N of neurons	

Transection basal	
1.5335	
1.25E-01		
Transection basal	- 2.2644	5.36E-
02	
2	
21, mouse1= 12, mouse2= 9	
transection day 1	1.4544	0.14583228		transection day 1	8.4497	0.092	2	20, mouse1= 11, mouse2= 9	

transection day 20	- 2.1696	
0.06003845		
transection day 20	- 4.9134	8.90E-
02	
2	
17, mouse1= 9, mouse2= 8	
									

Crush basal	
0.4264	
0.66985117		
Crush basal	
-0.332	7.40E-
01	
2	
24, mouse1= 12, mouse2= 12	

Crush day 1	
1.4544	
0.25		
Crush day 1	- 0.5536	5.80E-
01	
2	
22, mouse1= 12, mouse2= 10	

Crush day 20	- 7.1483	
8.74E-02		
Crush day 20	
2.4611	5.39E-
02	
2	
15, mouse1= 9, mouse2= 6	

Statistical details in population neuronal activity in ALM pre and post control events. Differences between z-score pre and post walking and activation of stepper motor in population neuronal activity of two mice. Significance level p<=0.05.
